# High-fidelity modular skeletons authenticate a Cambrian origin for Bryozoa

**DOI:** 10.1038/s41586-026-10590-9

**Published:** 2026-06-03

**Authors:** Baopeng Song, Zhifei Zhang, Luke C. Strotz, Timothy P. Topper, Andrej Ernst, Junye Ma, Zhiliang Zhang, Mei Luo, Lars E. Holmer, Yue Liang, Yazhou Hu, Caibin Zhang, Yanlong Chen, Glenn A. Brock

**Affiliations:** 1 State Key Laboratory of Continental Evolution and Early Life, Shaanxi Key Laboratory of Early Life and Environments, Department of Geology, Northwest University, Xi’an, China; 2 Department of Earth Sciences, Faculty of Geosciences, Utrecht University, Utrecht, The Netherlands; 3 Department of Palaeobiology, Swedish Museum of Natural History, Stockholm, Sweden; 4 Institut für Geologie, Universität Hamburg, Hamburg, Germany; 5 State Key Laboratory of Palaeobiology and Stratigraphy, Nanjing Institute of Geology and Palaeontology, Chinese Academy of Sciences, Nanjing, China; 6 Department of Earth Sciences, Palaeobiology, Uppsala University, Uppsala, Sweden; 7 School of Natural Sciences, Macquarie University, Sydney, New South Wales Australia

**Keywords:** Palaeontology, Sedimentology

## Abstract

The major animal body plans originated during the Cambrian explosion, yet the phylum Bryozoa has remained a conspicuous exception to this pattern^1^. The initial discovery of *Protomelission gatehousei*^2^ provided compelling evidence for a Cambrian origin for the Bryozoa, together with other major metazoan phyla and compatible with independent molecular clock estimates^3–7^. Nevertheless, the scarcity of definitive soft-tissue anatomy and diagnostic skeletal microstructure has left its phylogenetic affinities ambiguous and debated^8,9^. Here we report exquisite fossils of *P. gatehousei* and a new taxon, *Dayingomelission hexaclitia* gen. et sp. nov., from the early Cambrian Xiannüdong Formation of China. These specimens preserve in situ phosphatized soft tissues in modular skeletons, revealing critical anatomical structures, including styles, annular muscles, membranous sacs and ring septa. This suite of traits provides definitive evidence that these taxa belong to the Bryozoa. Phylogenetic analysis incorporating these new features identifies them as crown group stenolaemates. These results confirm a Cambrian origin for the phylum and reveal an unexpected early disparity in colonial architecture, demonstrating that bryozoan diversification was an integral component of the Cambrian radiation. Moreover, the early appearance of a differentiated stenolaemate crown group indicates a still deeper origin for the bryozoan stem lineage than was first apparent.

## Abstract (web summary)

Exquisite early Cambrian fossils of *Protomelission gatehousei* and *Dayingomelission hexaclitia* preserve soft-tissue anatomy and skeletal microstructure, confirming that these taxa are definitively bryozoans and indicating a deeper origin for the Bryozoa than was first apparent.

## Main

The origin of the colonial lophotrochozoan phylum Bryozoa has often been considered an evolutionary enigma, because a diverse fossil record comprising six of the eight recognized bryozoan orders appears suddenly in the Early Ordovician Epoch^10^. The timing of this event is in stark contrast to molecular clock analyses, which have consistently indicated an origin for bryozoans in the early Cambrian period (Terreneuvian Epoch)^3–6^ and is inconsistent with almost all other animal phyla, which first appeared during the Cambrian evolutionary radiation^6,7,11^. Several putative Cambrian bryozoans have been previously proposed (such as *Pywackia*^12,13^, *Archaeotrypa*^14^ and *Marcusodictyon*^15^) but have generally been discredited^16–20^.

Resolution of this conundrum seemingly came in the form of the modular, bilaminate colony of *P. gatehousei*, described from the lower Cambrian of South China and South Australia^2^ and recognized as the first well-supported candidate for a stem-group bryozoan on the basis of its mosaic of well-defined character traits shared with both gymnolaemates and mineralized stenolaemates. The bryozoan affinities of *P. gatehousei* have been widely accepted^21–26^, aligning the origins of the Bryozoa with other skeletonized clades and integrating bryozoans into the broader context of the Cambrian radiation. However, this interpretation has been subsequently challenged by alternative hypotheses that have relied on the absence of definitive bryozoan soft-tissue anatomy and diagnostic skeletal microstructure to suggest that the phylogenetic position of *P. gatehousei* may lie outside the Bryozoa^8,9^.

**Fig. 1:**
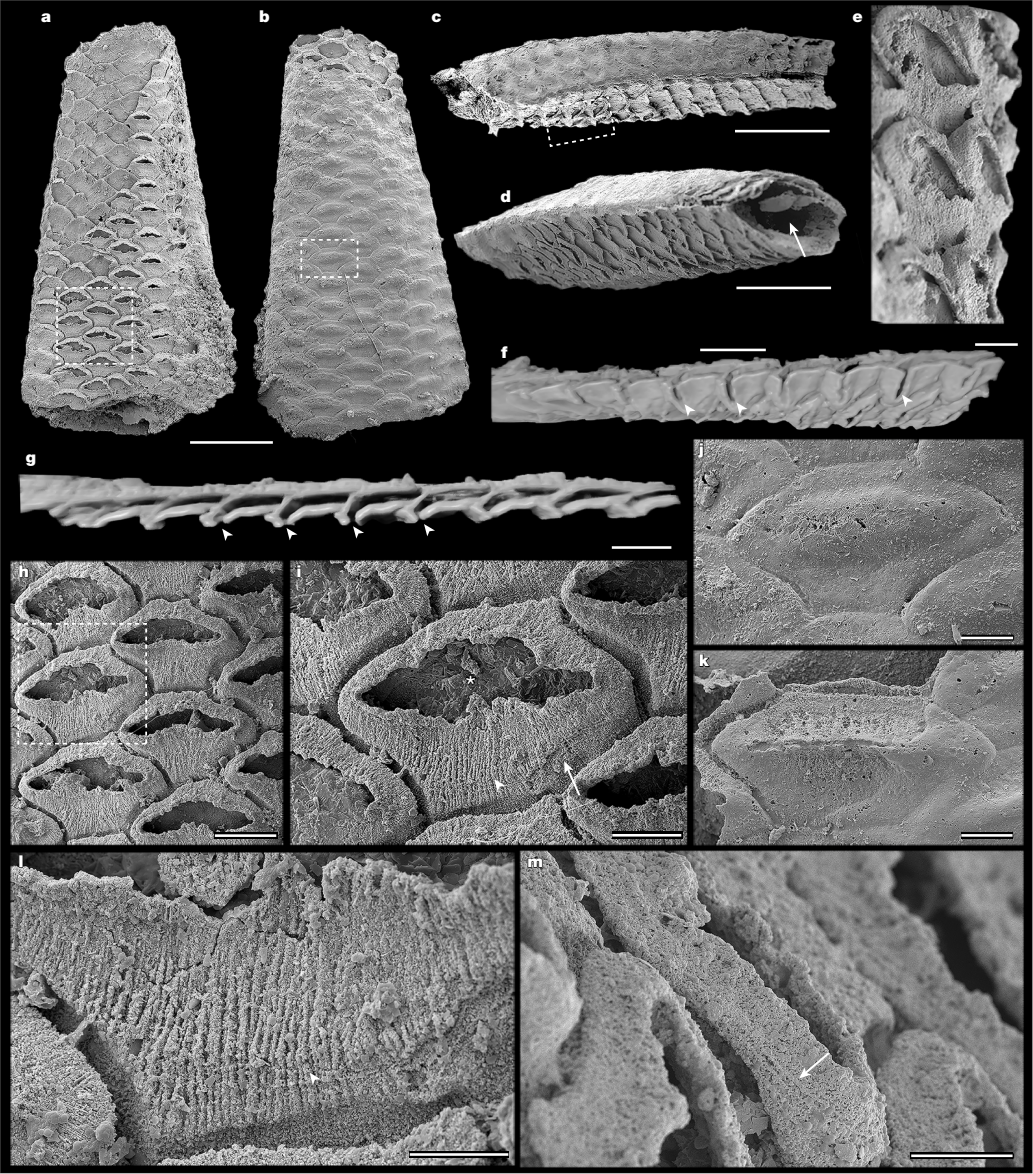
Specimen of *P. gatehousei* from the Xiannüdong Formation in which the membranous sacs are preserved (ELI DYCX 8-001). **a**, Front side of the colony. The outlined area is magnified in **h**. **b**, Back side of the colony. The outlined area is magnified in **j**. **c**, Lateral view of the bifoliate colony. **d**, Oblique lateral view of the bifoliate colony showing the hollow arched mesotheca (arrow). **e**, Partial enlargement of **c** showing the staggered budding pattern. **f**,**g**, X-ray tomographic microscopy images showing the longitudinal section of the colony and the orifice of autozooids (arrowheads) (**f**, oblique lateral view; **g**, lateral view). **h**, Quincuncial arrangement of sub-hexagonal membranous sacs with elliptical orifice. Note the 10-μm gap present between adjacent membranous sacs, indicating the loss of skeletal walls during the taphonomic processes. The outlined area is the membranous sac magnified in **i**. **i**, Enlarged view of a membranous sac showing the orifice (asterisk), circular fibres (arrow) and longitudinal fibres (arrowhead). These features suggest muscle preservation in the membranous sac. **j**, Enlarged view of a zooid. Note that the aperture is coated with secondary phosphate (the energy-dispersive spectroscopy analysis of this aperture is shown in Extended Data Fig. 5). **k**, Enlarged view of a zooid. Note that the secondary phosphate coating of the aperture is partially stripped away. **l**,**m**, Enlarged view of the membranous sac showing the longitudinal fibres (**l**, arrowhead) and circular fibres (**m**, arrow). These features suggest muscle preservation in the membranous sac. Scale bars, 500 μm (**a**–**d**), 50 μm (**e**,**i**–**k**), 200 μm (**f**), 150 μm (**g**), 100 μm (**h**) and 30 μm (**l**,**m**).

**Fig. 2:**
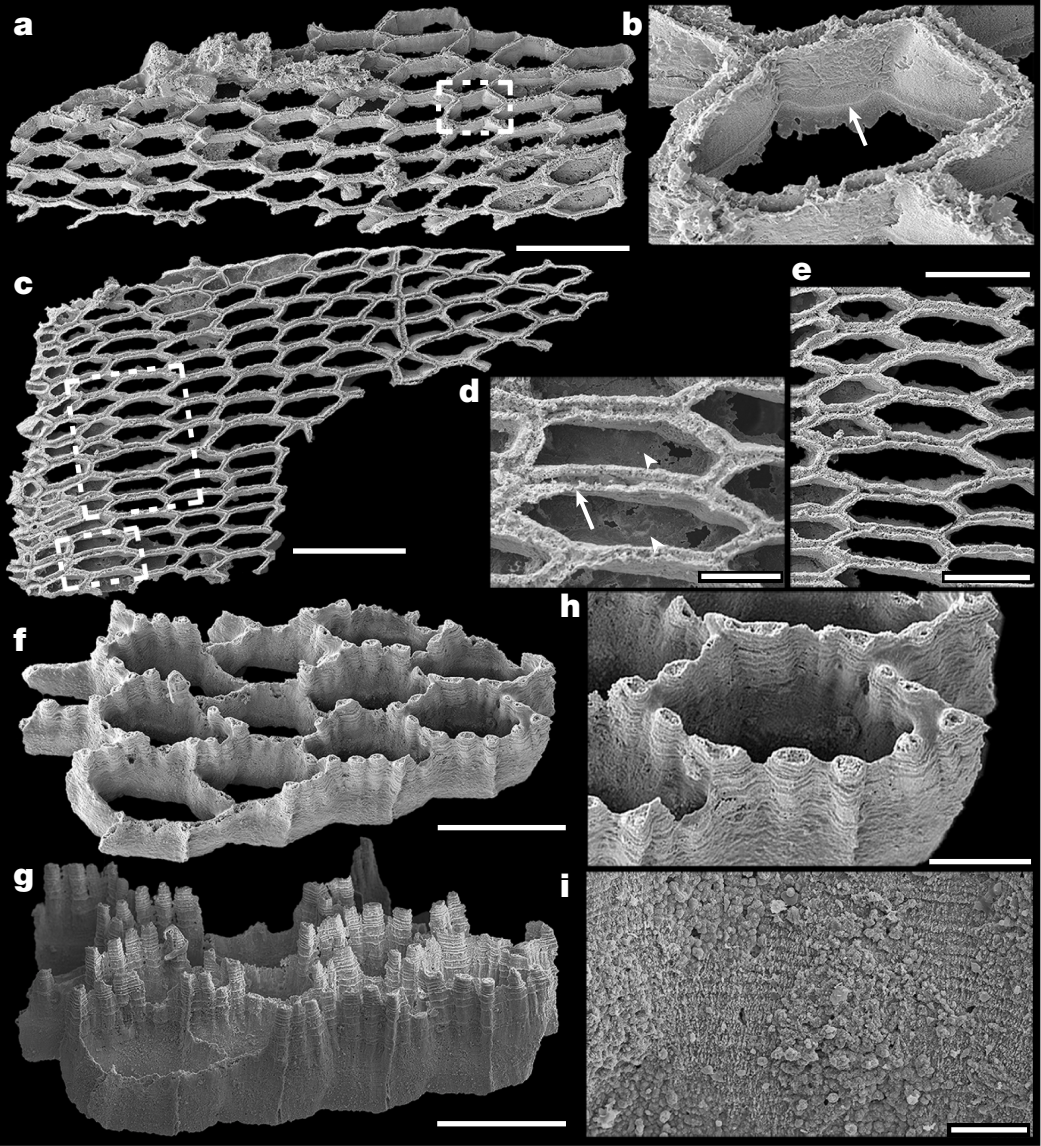
Specimens of *D. hexaclitia* gen. et sp. nov. from the Xiannüdong Formation showing the colony and cystids. **a**,**b**, ELI ZJBX 10-001 (holotype). **a**, Oblique view of the front side of a unilaminate colony form clearly showing the regular hexagonal, compactly arranged, honeycomb-shaped cystids. The outlined area is shown in **b**. **b**, Hexagonal cystid with vertical wall and ring septa clearly evident (arrow). **c**–**e**, ELI ZJBX 10-002. **c**, Front side of a unilaminate colony form. The bottom outlined area shows the cystids magnified in **d**; whereas the top outline shows the cystids magnified in **e**. **d**, Enlarged view of adjacent cystids. Note the hexagonal vertical wall (arrow) and the basal exterior wall of cystids (arrowheads). **e**, Row bifurcation showing change in zooid width along rows. **f**–**i**, ELI ZJBX 3-001. **f**, Front side of a unilaminate colony form with styles indenting the zooidal chambers. **g**, Oblique view showing hexagonal cystids with styles. **h**, Oblique view of colony surface. Note that the styles arise in the endozone and extend through most of exozone. **i**, Enlarged view of the vertical wall with planar spherulitic fabric. Scale bars, 500 μm (**a**,**c**), 80 μm (**b**), 100 μm (**d**), 200 μm (**e**), 300 μm (**f**,**g**), 100 μm (**h**) and 25 μm (**i**).

**Fig. 3:**
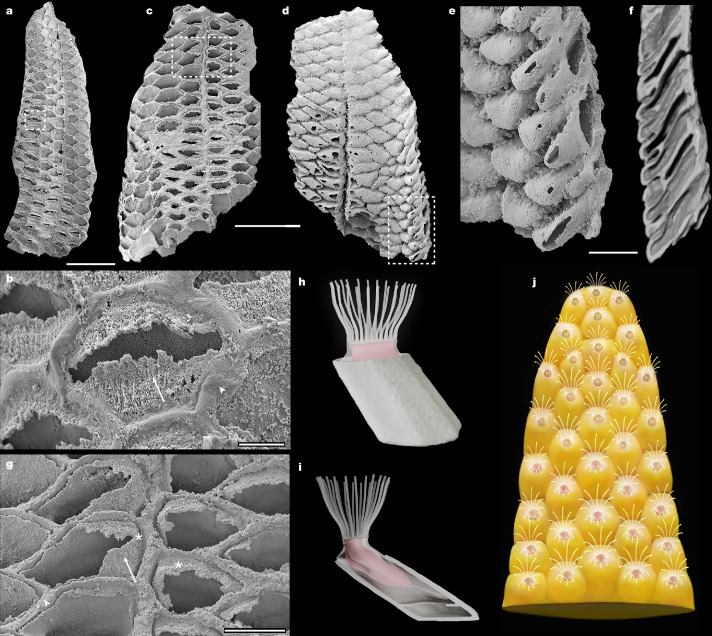
Membranous sacs preserved in situ in the autozooid cystids of *P. gatehousei* and *D. hexaclitia* gen. et sp. nov. and colonial growth reconstruction of *P. gatehousei.* **a**,**b**, *P. gatehousei* ELI DYCX 8-016. **a**, Front side of a bifoliate colony showing the eight series of zooids. The outlined area is magnified in **b**. **b**, Enlarged view of a zooid. Note that the membranous sac (arrow) is preserved in the cystid (arrowhead). **c**–**g**, *D. hexaclitia* ELI DYCX 8-004. **c**, Front side of a unilaminate colony, with ten series of zooids, all with membranous sacs and cystids. The outlined area is magnified in **g**. **d**, Back side of the colony showing the membranous sacs of the zooids and the gap between the sacs. The outlined area is magnified in **e**. **e**, Enlarged view showing capsule-like membranes and gaps. **f**, X-ray tomographic microscopy image showing the longitudinal section of zooids with membranous sacs and cystids. **g**, Enlarged view highlighting that the membranous sacs (arrow) are captured in the cystids (arrowhead), and the membranous sacs are in contact with the cystids 20 μm from the apertures (ligamentous attachment, asterisks). **h**, Three-dimensional reconstruction of a bryozoan zooid with protruding lophophore. **i**, Longitudinal section of reconstructed bryozoan zooid. Greyish white, cystid; translucent white, membranous sac and tentacles; pink, polypide excluding tentacles. **j**, Reconstruction of *P. gatehousei*, front surface view. Illustration in **j** adapted from ref. ^2^, under a Creative Commons licence CC BY 4.0. Scale bars, 500 μm (**a**,**c**,**d**), 40 μm (**b**), 200 μm (**e**,**f**) and 100 μm (**g**).

**Extended Data Fig. 1:**
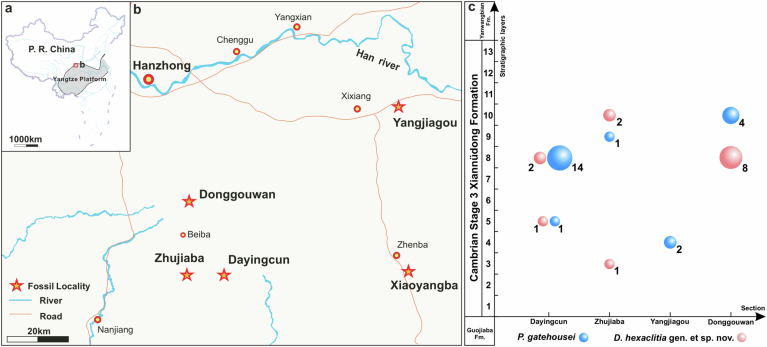
Geographic map of fossil localities. **a**, Map of China, modified from the Standard Map of China (No. GS (2023)2767). **b**, Locality details of the Zhujiaba, Dayingcun, Yangjiagou, Donggouwan and Xiaoyangba sections, southern Shaanxi Province, South China. The Xiaoyangba locality represents the lowest stratigraphic occurrence of *Protomelission gatehousei*^2^. **c**, Bubble diagram showing the distribution of specimens of *P. gatehousei* and *D. hexaclitia* gen. et sp. nov. in the Xiannüdong Formation stratigraphic succession.

Here we describe new specimens from the early Cambrian Xiannüdong Formation (southern Shaanxi, China; Extended Data Fig. 1), representing two bryozoan morphotypes: the modular bilaminate colony of *P. gatehousei* (Fig. 1) and a unilaminate colony ascribed to a new genus, *Dayingomelission hexaclitia* gen. et sp. nov. (Fig. 2). Crucially, these specimens preserve not only the skeletal traits previously used to establish the bryozoan affinities of *P. gatehousei* but also soft-tissue features of exceptional fidelity, including internal moulds of membranous sacs in the zooid chambers (Fig. 3). This combined set of characters unequivocally validates the assignment of these two taxa to the Bryozoa, reaffirming a Cambrian origin for the phylum. The presence of two distinct genera indicates that bryozoans were already diversifying during the Cambrian radiation.

**Extended Data Fig. 2:**
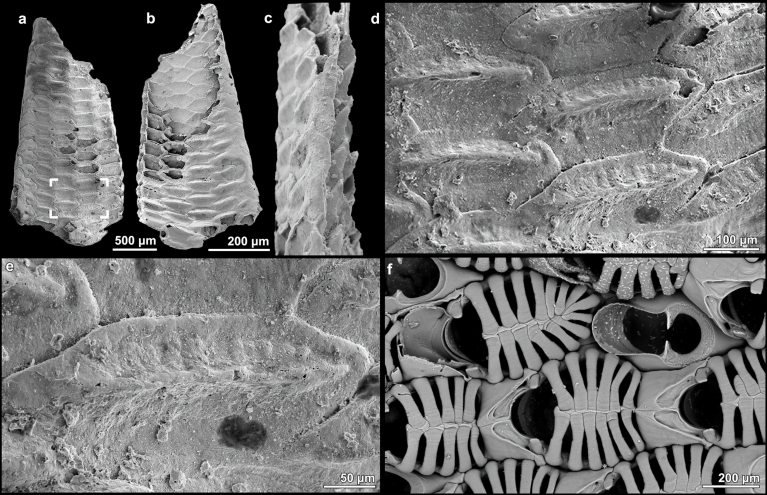
Soft-tissue preservation of *Protomelission gatehousei.* **a**–**e**, ELI DYCX 8-005. **a**, Front side of the colony, box corners indicate the area shown in **d**. **b**, The back side of the colony. **c**, Lateral view of the bifoliate colony. **d**,**e**, Enlarged view of elongated hexagonal zooids. Note the longitudinally neatly arranged cylindrical structures on the both sides of the ridge-like orifice, which are possible secondary coatings of protective shields. **f**, Protective shields developed in an extant cheilostome *Valdemunitella* sp. photographed by Dennis Gordon (Wellington).

**Extended Data Fig. 3:**
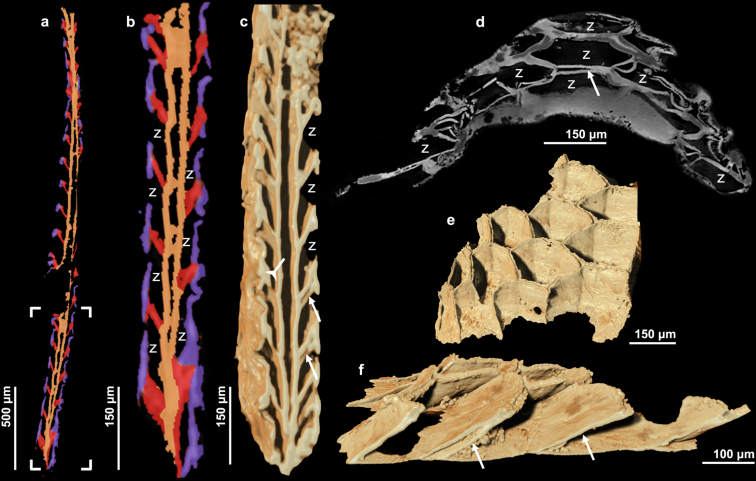
X-ray tomographic microscopy images of *Protomelission gatehousei* and *Dayingomelission hexaclitia* gen. et sp. nov. **a**–**c**, *P. gatehousei*. ELI DYCX 8-016. **a**,**b**, CT-reconstructed longitudinal section with false-colour delineating the distribution of the frontal surface (purple), vertical wall (red) and basal wall (yellow). Boxed region in **a** indicates the areas enlarged in **b** and **c**. **c**, Enlarged CT-reconstructed longitudinal section rendered using a bone colour palette. **d**, ELI DYCX 8-005, transverse section of *D. hexaclitia* gen. et sp. nov., note the regular hexagonal compactly arranged cystids in original greyscale. **e**,**f**, *P. gatehousei* rendered using a bone colour palette. ELI DYCX 8-013. **e**, Front side of colony, showing the hexagonal cystids with vertical walls. **f**, longitudinal section of **e**, showing phosphatized skeleton preserved in high fidelity (in contrast to other specimens, e.g. Extended Data Fig. 6) and indicating the presence of a double wall. Z, zooids; tailed arrow, mesotheca; arrows, vertical walls.

A collection of 38 modular fossils (see Methods section ‘Materials and occurrences’ for a full catalogue of specimens) are preserved as millimetric, secondarily phosphatized colonies (Figs. 1a–d, 2a–c,f–g and 3a–d). *P. gatehousei* has an erect, bilaminate, oligoserial colony form, 1–2 mm in width and up to 3 mm in height, tapering distally (Figs. 1a–d and 3j and Extended Data Fig. 2a–c). Each colony consists of two curved sheets of zooids arranged back to back, comprising six to eight rows of autozooids per side, with budding originating from a planar mesotheca (Extended Data Fig. 3a–c) or a flattened axial cylinder (Fig. 1d). *D. hexaclitia* is characterized by a unilaminate colony (Fig. 2a,c) (see ‘Systematic palaeontology’ section). Its autozooid chambers align within a single plane, growing almost perpendicular to the basal exterior wall (Fig. 2c–e). The colony surface displays a distinct zooidal arrangement, with colony growth proceeding through bifurcation (Fig. 2e). One of the unilaminate specimens (ELI ZJBX 3-001) preserves elongated, elliptical–cylindrical styles that originate in the endozone and extend prominently throughout the exozone (Fig. 2f–h).

**Extended Data Fig. 4:**
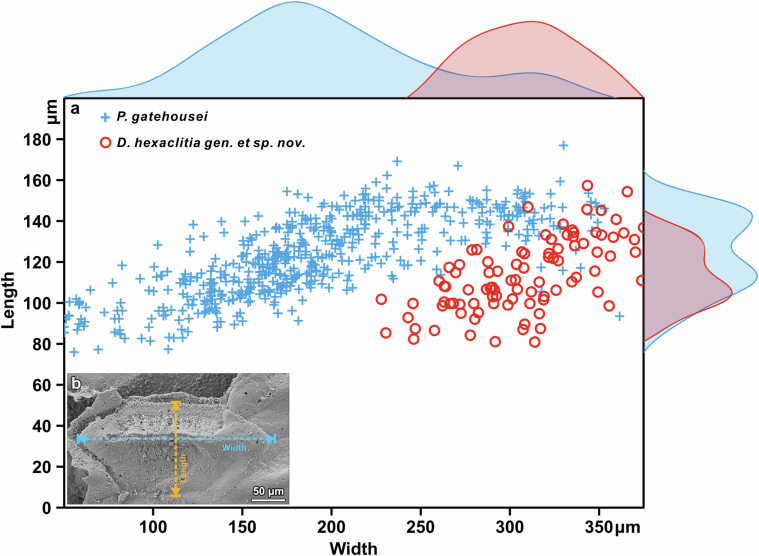
Scatter and violin plots of zooid size of *Protomelission gatehousei* and *Dayingomelission hexaclitia* gen. et sp. nov. Note in **a** that *P. gatehousei* and *D. hexaclitia* gen. et sp. nov. show distinct differences in zooid width. Values for boxplots are provided in Supplementary Data 1. N = 646 independent measurements of zooid size. Zooid length and width are defined in **b** (ELI DYCX 8-001). Length refers to the longitudinal axis of zooids (parallel to the growth axis), while width refers to the transverse axis measured perpendicular to the growth direction.

All zooids of both taxa are hexagonal in outline and box-shaped in profile, with a phosphatized or silicified skeleton (cystid) present in all colony forms (Fig. 2a–e and Extended Data Fig. 3a–c). On the frontal surface, these autozooids have an average width of 208.4 μm and a length of 121.7 μm (Extended Data Fig. 4 and Supplementary Data 1). The cystids are uniform in size, short and box-shaped, angled at 30–75° to the median lamina (mesotheca) (Extended Data Fig. 3a–c,f) or basal exterior wall (Fig. 2d). The skeleton is composed of non-porous basal and vertical walls. Although the vertical walls of adjacent zooids form a double wall (Fig. 2a–e and Extended Data Fig. 3d–f), the frontal (exterior) skeleton walls are structurally absent. The internal cystid walls of *D. hexaclitia* have a planar spherulitic fabric (Fig. 2i), whereas its vertical walls preserve ring septa (Fig. 2b).

**Extended Data Fig. 5:**
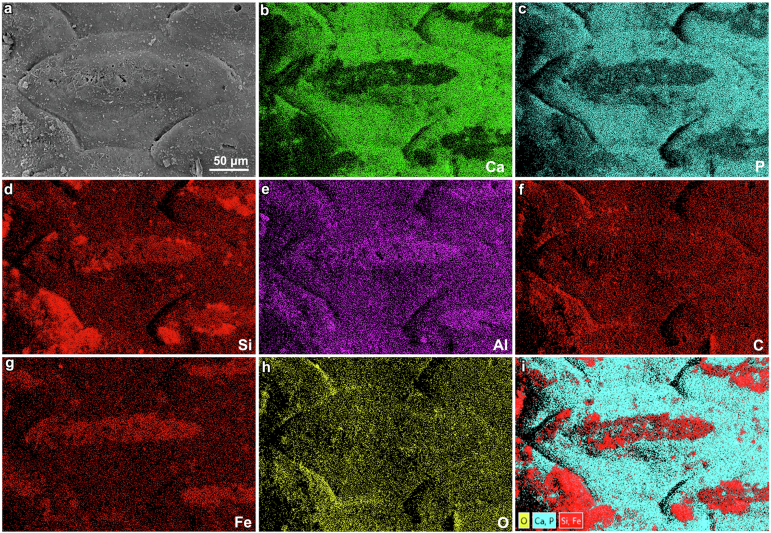
Elemental mapping of the zooids of *Protomelission gatehousei* in Fig. 1j, showing the orifice is coated by secondary phosphate, ELI DYCX 8-001. **a**, SEM image of zooids. **b**, Elemental map of Ca. **c**, Elemental map of P. **d**, Elemental map of Si. **e**, Elemental map of Al. **f**, Elemental map of C. **g**, Elemental map of Fe. **h**, Elemental map of O. **i**, Elemental map of O, Ca and P, Si and Fe concentrations, noting the orifice is filled with clay minerals.

**Extended Data Fig. 6:**
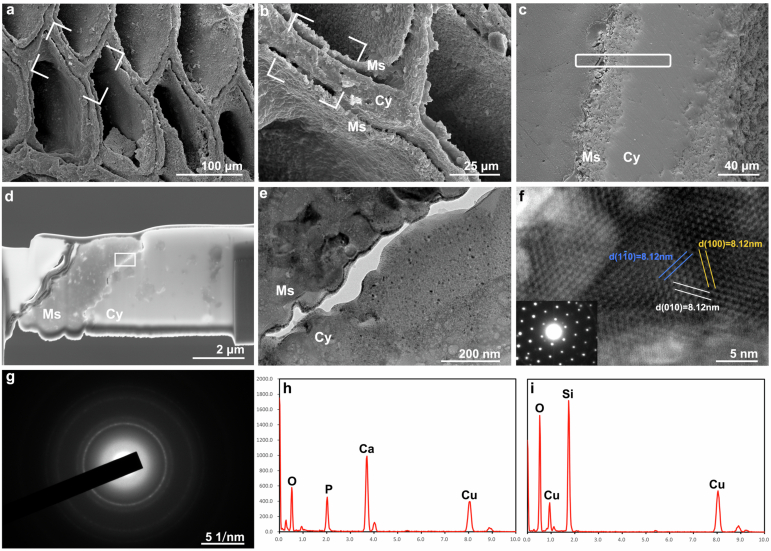
TEM characterization of the mineralogy and chemistry of the vertical skeletal wall and its relationship with the preserved membranous sacs in *Dayingomelission hexaclitia* gen. et sp. nov., ELI DYCX 8-004. **a**, Enlarged view of **c** in Fig. 3, **b**, Enlarged view of **a**, showing the cystids and membranous sacs at the boundary of the three zooids, box corners indicate the area shown in **c**. **c**, SEM image after encapsulating and polishing to the test exposure surface, note the cystids are compact and no particles visible, sac consists of small particles tightly packed. White box showing FIB cutting area. **d**, SEM image of the FIB ultra-thin foid sample prepared by FIB-SEM, white box indicates the area shown in **e**. Note the apatite particles are embedded in cystids. **e**, HAADF image of FIB ultra-thin foid, showing no obvious replacement relationship between cystid and membranous sac, cystids consist of amorphous SiO_2_ and membranous sac consists of crystalline apatite nanoparticles. **f**, HRTEM image of apatite nanoparticle in membranous sac, with Miller indices and the corresponding d-spacing values, bottom left corner indicates SAED pattern of apatite nanoparticles in membranous sac. **g**, SAED pattern of cystid, revealing its amorphous silicon composition. **h**,**i**, EDXS spectra of membranous sac (**h**) and cystid (**i**) derived from the microregions (<50 nm) in **d**, respectively. Cy, cystid; Ms, membranous sac.

**Extended Data Fig. 7:**
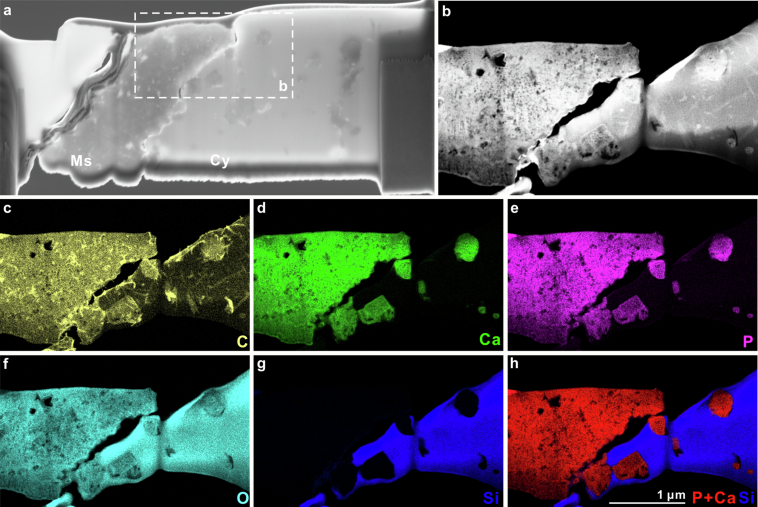
STEM-EDXS elemental maps of cystid and membranous sac of *Dayingomelission hexaclitia* gen. et sp. nov., ELI DYCX 8-004. **a**, SEM image of the FIB ultra-thin foil sample prepared by FIB-SEM, white box indicates the STEM-EDXS elemental map area. Cy, cystid; Ms, membranous sac. **b**, STEM-HAADF image of elemental map area. **c**, Elemental map of C. **d**, Elemental map of Ca. **e**, Elemental map of P. **f**, Elemental map of O. **g**, Elemental map of Si. **h**, Elemental map of P, Ca and Si.

The zooids of *P. gatehousei* and *D. hexaclitia* contain phosphatized structures interpreted as membranous sacs, featuring thin walls and smooth outlines (Figs. 1a,e,h,i and 3b,g). The distal end of each membranous sac consists of a long, elliptical orifice surrounded by an undulating fold (Fig. 1h,i and Extended Data Fig. 5i). A consistent, uniform, 10–20-µm-wide gap separates adjacent sacs (Fig. 1h,i). The densely packed, well-developed circular and longitudinal fibres on the membranous sac surfaces represent annular and longitudinal muscles (Fig. 1i,l,m). The sac membranes are composed of crystalline apatite particles (Extended Data Figs. 6 and 7). In ELI DYCX 8-001, the phosphatized sacs and secondarily filled orifices (Extended Data Fig. 5) are overgrown by a subsequent phosphate coating, forming smooth bulges (Fig. 1b,j,k), indicating a multiphase phosphatization history. Longitudinally aligned cylindrical structures, possibly representing protective shields or a broad operculum, are preserved on both sides of the secondary orifice coatings (Extended Data Fig. 2a–f). The exceptional preservation of specimens ELI DYCX 8-016 and ELI DYCX 8-004 reveals that the membranous sacs are preserved in situ in the autozooid cystids (Fig. 3a–c,g–j). The sac is attached to the cystid wall in the distal apertural area, with a consistent spacing of 10–20 µm (Fig. 3g), matching the thickness interval of adjacent sacs in specimen ELI DYCX 8-001 (Fig. 1h,i). Transmission electron microscopy confirms the absence of a replacement relationship between the phosphatized sac and the silica-replaced cystid wall in ELI DYCX 8-004 (Extended Data Fig. 6), indicating their independent preservation pathways.

## Discussion

**Fig. 4:**
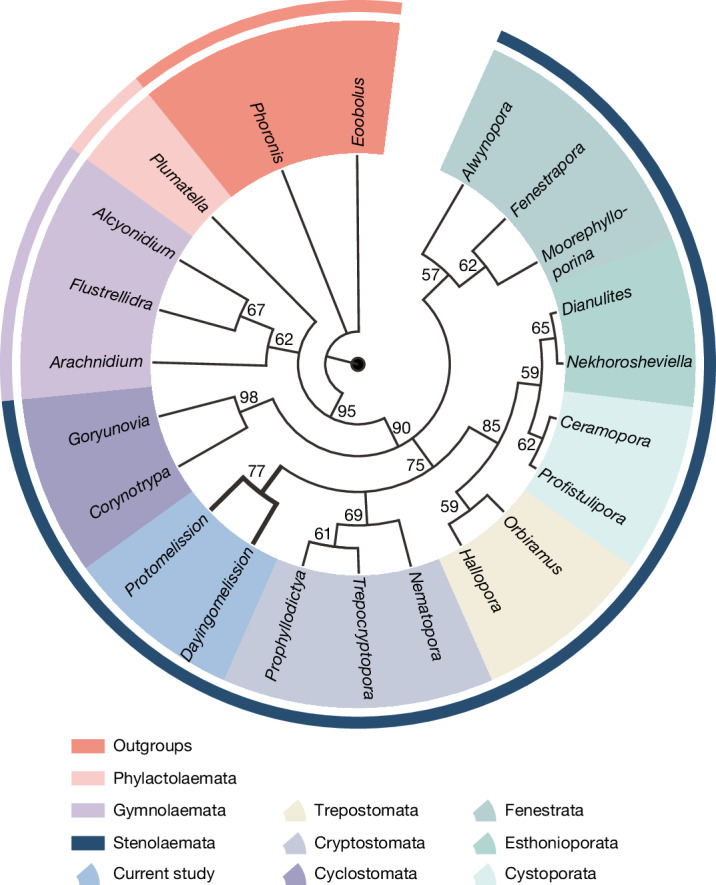
Phylogenetic relationships of bryozoans. A 50% majority-rule consensus phylogenetic tree inferred using morphological characters and Bayesian analysis based on a matrix of 22 taxa and 50 characters (see Methods and Supplementary Data 2–4 for source data and further information). Node values are Bayesian posterior probability support values. Coloured areas indicate the three taxonomic classes that comprise the Bryozoa along with outgroups, with *Protomelission* and *Dayingomelission* belonging to Stenolaemata.

*P. gatehousei* and *D. hexaclitia* satisfy almost all key diagnostic criteria for Palaeozoic bryozoans and share several traits with Class Stenolaemata^27–29^ (Extended Data Table 1). These shared traits include aspects of colony morphology^30–32^ (Fig. 1a,b and Supplementary Videos 1–6), skeletal architecture^10,29,32,33^ (Fig. 2 and Extended Data Fig. 3) and the presence of soft-tissue structures (Fig. 1 and Extended Data Fig. 2), such as membranous sacs^24,28^ (Fig. 1h), as well as annular and longitudinal musculature (Fig. 1i,l,m). Crucially, assignment to the Stenolaemata is strongly supported by the presence of several apomorphic palaeostomate features, including styles^32,34^ (Fig. 2f–h) and a free-walled colony organization^10,35^ (Figs. 2 and 3). A phylogenetic analysis incorporating these new traits now robustly identifies these early Cambrian forms both as crown group bryozoans and as part of the Stenolaemata (Fig. 4 and Supplementary Data 2–4). This pivotal revision moves beyond the previous stem-group hypothesis for *P. gatehousei*^2^. With both Cambrian bryozoan taxa now considered to be stenolaemates, they were conceivably biomineralized^36^, although on the basis of the available evidence (Extended Data Figs. 5–7) their primary skeletal composition remains unknown. This establishes the possibility that the first of several independent biomineralization events in the Bryozoa^36^ occurred during the early Cambrian, a scenario consistent with molecular analyses^23,37^. These results also imply that the common ancestor of the organic-walled Gymnolaemata and the mineralized Stenolaemata probably originated in the early Cambrian (Terreneuvian) or even perhaps in the Ediacaran period^21,23,38^.

These newly described specimens directly address and refute alternative interpretations that have suggested *P. gatehousei* should not be considered a bryozoan. A previous study^8^ proposed that *P. gatehousei* represents a dasycladalean green alga, relying heavily on comparisons with larger, highly compressed Xiaoshiba fossils. These specimens differ critically from both the original specimens of *P. gatehousei*^2^ and our own material. First, *P. gatehousei* specimens are small (2–3 mm), phosphatized, three-dimensional and bilaminate, with a tapering outline and a regular hexagonal chamber arrangement^2^ (Fig. 1a–d and Extended Data Table 1). By contrast, the Xiaoshiba fossils are more than 20 times larger, clavate and have a single-layer wall^8^. Second, *P. gatehousei* possesses a suite of definitive bryozoan traits absent in the Xiaoshiba material, including zooidal budding from an endozone that produces overlapping zooids at an acute angle to a well-defined mesotheca^2,39^, (a characteristic commonly seen in ptilodictyine bryozoans^40^; Extended Data Fig. 3). Conversely, the Xiaoshiba modules have non-overlapping units and a dual-aperture structure more similar to archaeocyath bracts and pores^41^. These fundamental discrepancies indicate that the Xiaoshiba fossils are fundamentally different and unrelated to *P. gatehousei*. The alternative hypothesis that *P. gatehousei* represents the sclerites of *Cambroclavus*^9^ is also inconsistent with our evidence. In complete specimens of *P. gatehousei*, the zooidal aperture either has a distinct, elongated–elliptical orifice (Fig. 1h,i) or is occluded by secondary phosphatic coatings, with only residual clay minerals marking its original position (Fig. 1b,j, Extended Data Fig. 5 and Supplementary Videos 1 and 2). By contrast, *Cambroclavus* is characterized by isolated sclerites with solid, circular spines in cross-section. *Cambroclavus* sclerites preserved in the mesotheca-subtended cylindrical cavities of *P. gatehousei* are disordered infills, not in situ structures^2,9^. Both anatomical and taphonomic evidence thus unequivocally establish that *P. gatehousei* and *D. hexaclitia* represent Cambrian bryozoans.

The presence of two bryozoan taxa reported here from the Xiannüdong Formation along with *P. gatehousei* from the lower Wirrealpa Limestone of South Australia^2^ and a possible mineralized bryomorph from the lower Cambrian of Nevada^22^ indicates that Cambrian bryozoans were more prevalent and widespread in early Cambrian shelf seas than previously assumed^10,35^, perhaps particularly so in archaeocyath reef-associated carbonate platform settings^42^. The exquisite preservation of these taxa not only validates their bryozoan affinities but also reveals an unexpected early disparity in colony architecture. Furthermore, the exceptional preservation of soft tissues alongside skeletal structures provides critical morphological evidence for reconstructing the evolution of colonial complexity in metazoans^24,29,32^ and, combined with evidence for other metazoan clades^43–45^, highlights that hierarchical colonial organization evolved as a fundamental innovation during the Cambrian radiation.

## Systematic palaeontology

Phylum Bryozoa Ehrenberg, 1831

Class Stenolaemata Borg, 1926

Order and Family uncertain

*Dayingomelission* gen. nov. Song, Zhang & Ernst

**Diagnosis.** The colony is encrusting and unilaminate. Autozooecia are short and box-shaped, with hexagonal apertures. Vertical walls are straight or slightly inclined proximally. Adjacent autozooecia are separated by double-walled structures. Ring septa are present. Basal wall is planar to gently curved. Styles are occasionally present.

**Type species.** *D. hexaclitia* gen. et sp. nov. Song, Zhang & Ernst (Figs. 2a–i and 3c–g and Extended Data Fig. 6a,b).

**Etymology.** The generic name *Dayingomelission* combines the locality Daying with the Greek *melission* (honeycomb), alluding to the structure of the colony.

*D. hexaclitia* gen. et sp. nov. Song, Zhang & Ernst.

**Holotype.** ELI ZJBX 10-001 (Fig. 2a,b).

**Paratypes.** ELI ZJBX 10-002 (Fig. 2c–e), ELI DYCX 8-004 (Fig. 3c–g) and ELI ZJBX 3-001 (Fig. 2f–i).

**Etymology.** The species epithet *hexaclitia* is derived from the Greek *hexa* (six) and *klitia* (slope), describing the sloped, hexagonal apertures of the autozooids.

**Diagnosis.** Genus diagnosis is by monotypy.

**Description.** The colony is unilaminate, forming a sheet-like growth on the substrate (Fig. 2a,c). The available specimens are incompletely preserved, with the largest fragment exceeding 4 mm in maximum dimension and comprising more than ten zooidal rows (Fig. 2a). Astogeny occurs through linear budding, with branching observed where a single row of zooids diverges to form two new rows. Autozooids are short and box-like, arranged in a regular pattern (Fig. 2b). Their apertures are distinctly hexagonal, ranging from 200 µm to 400 µm in diameter (Extended Data Fig. 4). The orifice is incompletely preserved. The morphology of autozooids may show local deformation or reduction in size, probably resulting from spatial constraints during growth (Fig. 2c). The vertical walls are straight to very slightly inclined in the proximal direction. Adjacent autozooids are separated by a double-walled structure. The vertical walls are double-layered throughout (Fig. 2a–e). They are characterized by a planar spherulitic fabric in the endozone and bear 12–15 styles per autozooid aperture in the exozone of some specimens (Fig. 2f–h). These styles exhibit an elliptical cross-section, with the long axis measuring approximately 50 µm. Transverse ridges are developed on the vertical walls in the exozone (Fig. 2g). Internally, ring septa are consistently present (Fig. 2b). The basal wall is predominantly planar (Fig. 2i), with an occasional gentle curvature observed in some specimens.

**Remarks.** *D. hexaclitia* and *P. gatehousei* have similar hexagonal zooid apertures, and the arrangement of zooecial series is oligoserial in both taxa. In *P. gatehousei*, this oligoserial arrangement is expressed as a bilaminate colony with distally tapering frontal surfaces, which is a common growth pattern among early stenolaemate bryozoans, particularly in certain cryptostome lineages. However, *D. hexaclitia* can be clearly distinguished from *P. gatehousei* by its unilaminate colony arrangement (Figs. 1 and 2) and its larger aperture width (Extended Data Fig. 4). Any differences between the orifices remain unknown owing to the incomplete preservation of this feature in *D. hexaclitia*. The combination of a unilaminate colony, short box-like autozooids with hexagonal apertures and a distinctive double-walled structure confirms the bryozoan affinities of *D. hexaclitia* and serves to clearly distinguish it from other known bryozoan genera. The simple morphology of *D. hexaclitia*, particularly the lack of extra internal structures such as hemisepta, suggests a phylogenetic position among the early-diverging bryozoan groups.

The taphonomy of early Cambrian fossils preserved in carbonates is governed by complex, differential mineralization processes. In *D. hexaclitia*, for example, preservation varies from exclusively phosphatized skeletal walls (Fig. 2) to a mosaic of phosphatized membranous sacs and silicified skeletal walls (Fig. 3c–g). Conversely, exceptionally preserved *P. gatehousei* specimens exhibit robustly phosphatized soft tissues despite the near-total loss of vertical skeletal walls (Fig. 1h,i). Although such preservational biases, probably driven by localized geochemical microenvironments during diagenesis, can obscure vertical skeletal walls, their presence in both Cambrian bryozoan taxa is unequivocally supported by specimens in which soft tissues remain in situ in distinct skeletal chambers (Fig. 3b,g).

**Terminology.** In accordance with established bryozoan literature^29^, we define orifice as referring to the opening bordered by soft tissues (the membranous sac opening), and aperture refers to the skeletal opening.

**Stratigraphic occurrence.** The specimens originate from the Xiannüdong Formation, Cambrian Stage 3, southern Shaanxi, China. A total of 14 specimens were examined, with the following specimen numbers: ELI ZJBX 10-001–002, ELI ZJBX 3-001, ELI DYCX 5-002, ELI DYCX 8-004, ELI DYCX 8-015, ELI DGWX 10-020, ELI DGWX 10-022, ELI DGWX 10-023, ELI DGWX 10-025, ELI DGWX 10-030, ELI DGWX 10-031, ELI DGWX 10-046 and ELI DGWX 10-048.

## Methods

### Terminology

We adopted the morphological terminology used in previous studies of fossils and extant bryozoans^2,10,33,34,46,47^.

### Materials and occurrences

All fossils described in this study were collected from bioclastic and reef limestone outcrops of the Xiannüdong Formation (Cambrian Stage 3) in southern Shaanxi, South China. Carbonate rock samples were immersed in 5–10% acetic acid and washed twice weekly until all matrix material was completely dissolved, with insoluble residues collected after each treatment. Specimens were then picked from the washed and dried residues under a ZEISS binocular stereo microscope (Stemi 305). Measurements of fossil length, width and angle were obtained from X-ray computed tomography and scanning electron microscopy (SEM) images using ImageJ v.1.54f. Of the specimens, 19 are from the Dayingcun Section (ELI DYCX 5-001–002, ELI DYCX 8-001–016 and ELI DYCX 9-001), 3 are from the Zhujiaba Section (ELI ZJBX 3-001 and ELI ZJBX 10-001–002), 2 are from the Yangjiagou Section (ELI XX 4-001–002) and 14 are from the Donggouwan Section in Hanzhong, southern Shaanxi, South China (ELI DGWX 10-013, ELI DGWX 10-020, ELI DGWX 10-022, ELI DGWX 10-023, ELI DGWX 10-025, ELI DGWX 10-030, ELI DGWX 10-031, ELI DGWX 10-043, ELI DGWX 10-046, ELI DGWX 10-047, ELI DGWX 10-048, ELI DGWX 10-049, ELI DGWX 10-051 and ELI DGWX 10-052) (Extended Data Fig. 1b,c). The geological and geographical setting has been previously described in detail^42,48^. Among these specimens, those assigned to *P. gatehousei* include ELI DYCX 5-001, ELI DYCX 8-001–003, ELI DYCX 8-005–014, ELI DYCX 8-016, ELI DYCX 9-001, ELI XX 4-001–002, ELI DGWX 10-013, ELI DGWX 10-049, ELI DGWX 10-051 and ELI DGWX 10-052. Those classified as *D. hexaclitia* include ELI ZJBX 10-001–002, ELI ZJBX 3-001, ELI-DYCX 5-002, ELI DYCX 8-004, ELI DYCX 8-015, ELI DGWX 10-020, ELI DGWX 10-022, ELI DGWX 10-023, ELI DGWX 10-025, ELI DGWX 10-030, ELI DGWX 10-031, ELI DGWX 10-046 and ELI DGWX 10-048, with two indeterminate specimens ELI DGWX 10-043 and ELI DGWX 10-047. All specimens are deposited at Northwest University (NWU).

### SEM

The well-preserved specimens were gold-coated and examined using SEM at the State Key Laboratory of Continental Evolution and Early Life at NWU (model and parameters: FEI Quanta 450-FEG with 20.0 kV, 60 Pa and working distance of 8–10 mm; Phenom XL G2 with 5–15 kV, high vacuum mode and working distance of 6–8 mm). Further semi-quantitative analyses were performed using the energy spectrometer attached to the above SEM system.

### Focused ion beam–transmission electron microscopy

The fossils were directionally encapsulated by epoxy filling under vacuum and polished using a Cross Section Polisher (SEMPrep2 with 12.0 kV, 4.2°–4.4°, 1.09 × 10^4^ mbar and 3.8 mA) until the test surface was exposed. Ultrathin foils (15 μm in length, 10 μm in width and less than 100 nm in thickness) were prepared using the ZEISS Auriga Compact dual-beam focused ion beam–SEM (FIB–SEM) instrument equipped with an OmniProbe 200 micromanipulator (high voltages, 5–30 kV; beam currents, 50 pA–2 nA) at the Institute of Geology and Geophysics, Chinese Academy of Sciences (IGGCAS). High-angle annular dark-field imaging, selected area electron diffraction imaging and scanning transmission electron microscopy–energy dispersive X-ray spectroscopy (STEM–EDXS) mapping were performed using the JEOL JEM-2100 transmission electron microscope at the IGGCAS and the JEM-F200 at NWU.

### X-ray computed tomography

Five well-preserved specimens were scanned using a ZEISS Xradia CrystalCT, with the source operating at 80 kV and 125 µA over a 180° sample rotation (−92° to 92°) at NWU. The X-ray attenuation coefficients for each voxel were calculated from the scanned information and arranged into a digital matrix to reconstruct the image. X-ray computed tomography images were visualized and segmented through thresholding using ORS Dragonfly v.2022.2.0.1367. To help with differentiation, the morphological characteristics of interest were coloured differently from one another. Supplementary Videos 1–6 provide three-dimensional information (ELI DYCX 8-001, ELI DYCX 8-004, ELI DYCX 8-005, ELI DYCX 8-013 and ELI XX 4-001).

### Phylogenetic analysis

Fifty characters were coded for *Protomelission* and *Dayingomelission*, 18 bryozoan genera and 2 outgroup genera (a total of 22 genera). The resulting phylogenetic data matrix was analysed using the same methods outlined in previous work^2^. The 18 bryozoan genera are exemplars of the eight major bryozoan orders, and the fossil genera chosen are all from the Ordovician period (except for *Fenestrapora*, which is from the Devonian period). The two outgroup taxa (*Eoobolus* and *Phoronis*) correspond to the two major non-bryozoan clades in the Lophophorata. Character codings were based on previously published data (Supplementary Data 2–4). All character codings are provided in NEXUS format, along with a full list of the characters used in Supplementary Data 2–4. Phylogenetic trees were inferred using Bayesian methods. Bayesian analyses used MrBayes (v.3.2.7) with the Mkv model, incorporating gamma-distributed rate variation and variable coding. This analysis featured a sampling frequency of 1,000, two concurrent runs and four Metropolis-coupled chains. It was conducted over ten million generations. A 25% relative burn-in was applied for all summary statistics, and the resulting phylogenetic tree is shown in Fig. 4.

### Statistics and reproducibility

The SEM images and micrographs presented in this study were selected from all available materials to best illustrate the diagnostic features described. As this is a palaeontological study, it uses unique fossil specimens that are part of the evolutionary record, and, therefore, the exact fossils cannot be replicated. However, detailed locality information and complete methodological descriptions have been provided to enable independent resampling of the source horizons and repetition of all analytical procedures.

### Reporting summary

Further information on research design is available in the Nature Portfolio Reporting Summary linked to this article.

## Online content

Any methods, additional references, Nature Portfolio reporting summaries, source data, extended data, supplementary information, acknowledgements, peer review information; details of author contributions and competing interests; and statements of data and code availability are available at 10.1038/s41586-026-10590-9.

## Supplementary information

Supplementary Data 1–4 Supplementary Data 1 contains measurements of *P. gatehousei* and *D. hexaclitia* gen. et sp. nov. Supplementary Data 2 contains a character taxon matrix in NeXus format. Supplementary Data 3 contains character traits and morphological states for bryozoans. Supplementary Data 4 contains a character trait dataset matrix.

Reporting Summary

Peer Review File

Supplementary Video 1 Three-dimensional reconstruction of computed tomography data for ELI DYCX 8-001, showcasing the bilaminate colony of *P. gatehousei*.

Supplementary Video 2 Three-dimensional reconstruction of computed tomography data for ELI DYCX 8-001, with sections showing the details of the longitudinal sections of zooids and colony.

Supplementary Video 3 Three-dimensional reconstruction of computed tomography data for ELI DYCX 8-013, showing the double-walled structure of the colony on one side of *P. gatehousei*.

Supplementary Video 4 Three-dimensional reconstruction of computed tomography data for ELI DYCX 8-004, showcasing the unilaminate morphology of *D. hexaclitia* gen. et sp. nov. colony.

Supplementary Video 5 Three-dimensional reconstruction of computed tomography data for ELI DYCX 8-004, with sections showing the details of the longitudinal sections of membranous sacs.

Supplementary Video 6 Three-dimensional reconstruction of computed tomography data for ELI DYCX 8-005, showcasing the bilaminate morphology of *P. gatehousei* colony.

## Figures and Tables

**Extended Data Table 1:**
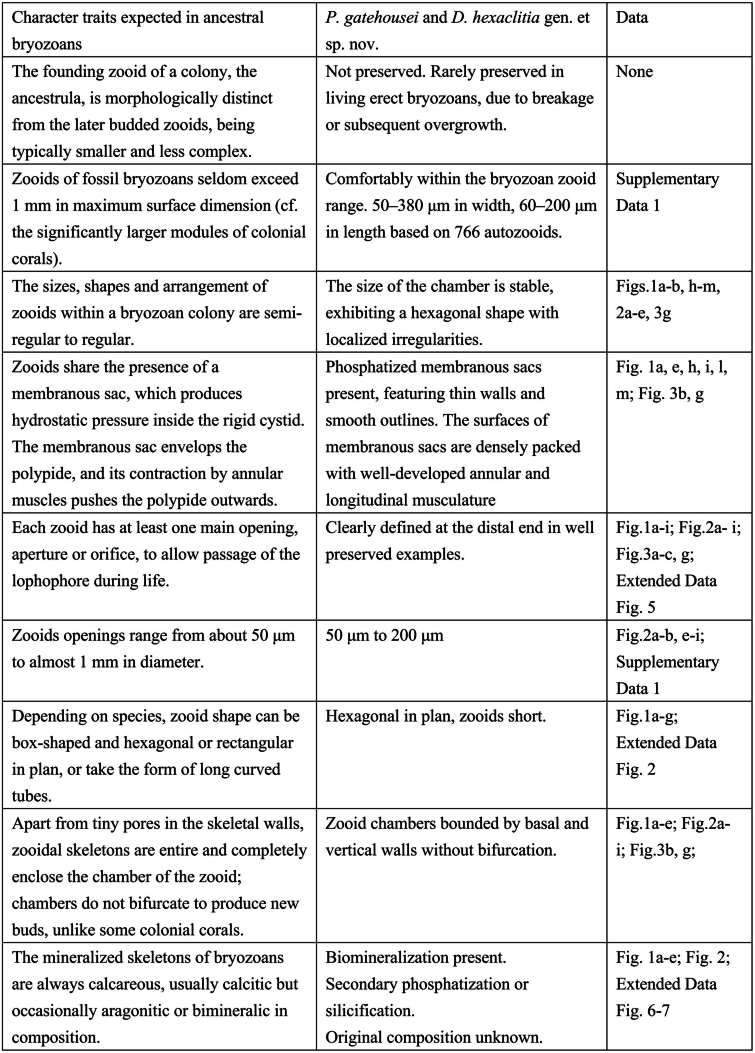
Character traits expected in ancestral Cambrian bryozoans

Adapted from ref. ^2^, Springer Nature Limited, under a Creative Commons licence CC BY 4.0.

## Data Availability

All data analysed in this study, including the phylogenetic datasets, are available within this paper and in Extended Data Figs. 1–7, Extended Data Table 1 and the Supplementary Information. Raw datasets are available at Figshare (10.6084/m9.figshare.31995942)^49^. Computed tomography scans of specimens in this publication can be accessed in the MorphoSource Repository (10.17602/M2/M851384, 10.17602/M2/M851389, 10.17602/M2/M851394, 10.17602/M2/M851399, 10.17602/M2/M851404 and 10.17602/M2/M851409) and the affiliated project (https://www.morphosource.org/projects/000850335?locale=en). The nomenclature of *D. hexaclitia* gen. et sp. nov. is registered in ZooBank (urn:lsid:zoobank.org:act:B5030E29-675D-4F12-9840-9999D64E52EB for genus and urn:lsid:zoobank.org:act:58073D95-64BE-4D1B-832C-1A5223E37837 for species). The Life Sciences Identifier for this publication is urn:lsid:zoobank.org:pub:D8979F9C-502B-41BE-9889-4510AB5664CD.
