# Supplementary material for: High-fidelity modular skeletons authenticate a Cambrian origin for Bryozoa
Source: Nature. 2026 Jun 3;656(8126):123–8. doi: 10.1038/s41586-026-10590-9 (PMC13441974; doi:10.1038/s41586-026-10590-9)
Supplement: Supplementary file 1 [file 41586_2026_10590_MOESM1_ESM.pdf]

nature portfolio

https://doi.org/10.1038/s41586-026-10590-9

Supplementary information

High-fidelity modular skeletons authenticate a Cambrian origin for Bryozoa

In the format provided by the authors and unedited

Supplementary Data 1 Measurement of *Protomelission gatehousei* and *Dayingomelission hexaclitia* gen. et sp. nov.All measurement units here are in micrometres (μm).

ELI-DYC-2-8-001A	Line1		Line2		Line3		Line4		Line5		Line6		Line7		Line8	
	Width	Length	Width	Length	Width	Length	Width	Length	Width	Length	Width	Length	Width	Length	Width	Length
1	236.802	169.172	216.173	145.653	197.061	184.032	175.003	154.472	226.772	151.582	177.944	139.703				
2	245.742	151.497	252.954	148.789	229.413	144.385			258.923	147.24	194.565	147.056				
3	213.232	149.997	257.365	157.357	233.819	148.556			245.624	154.521	220.898	144.235		139.703		
4	210.311	147.086	236.765	151.475	219.114	155.88			233.935	148.643	216.418	144.303	174.03	119.342		
5	200.083	141.205	211.781	142.652	219.114	153.193			226.586	147.064	218.14	150.35	200.261	147.24		
6	188.284	141.205	204.414	145.593	202.959	147.064			223.646	150.113	226.772	150.026	204.493	127.947	184.408	129.443
7	182.356	133.894	197.055	123.527	195.784	148.534			225.001	141.205	220.629	150.458	208.825	135.938	169.14	137.047
8	183.844	142.652	186.854	151.468	192.734	132.359			217.648	152.967	219.193	142.713	205.884	129.418	167.702	128.074
9	151.475	139.827	176.468	144.145	180.975	153.115			211.761	129.443	211.781	135.364	188.376	107.442	139.773	134.337
10	138.303	139.734	191.196	141.297	197.061	139.703			225.001	158.991	225.001	141.174	195.635	137.267	120.73	125.076
11	122.49	138.264	175.022	138.358	179.433	144.183			191.173	136.77	210.311	133.894	185.314	123.536	120.81	131.177
12	104.42	113.243	177.944	136.833	195.585	135.324			188.232	136.794	219.119	138.241	189.703	119.152	119.197	103.034
13	85.293	156.129	185.343	141.182	230.897	155.942					205.9	120.595	200.018	134.337	122.622	101.639
14			194.164	173.582	242.683	138.241					211.843	107.351	198.575	107.391	120.73	110.645
15			174.997	125.006	233.838	113.243					250.151	120.595	215.311	130.913	116.63	111.801
16					214.828						259.157	125.076	173.533	122.198		

ELI-DYC-2-8-001B	Line1		Line2		Line3		Line4		Line5		Line6		Line7	
	Width	Length	Width	Length	Width	Length	Width	Length	Width	Length	Width	Length	Width	Length
1			270.908	167.035	308.83	130.363	287.017	150.401	237.089	146.61	261.296	113.741	195.519	114.076
2	196.688	130.363	246.731	144.399	292.952	146.514	304.872	146.514	252.204	136.378	234.454	129.605	175.797	122.079
3	177.028	108.307	250.676	136.378	288.77	138.384	282.754	148.409	233.862	140.389	227.808	119.28	165.365	116.793
4	186.508	140.374	262.976	134.373	296.852	142.436	278.772	146.39	209.028	142.436	224.491	128.921	167.215	125.057
5	168.461	114.322	248.865	134.373	296.791	146.39	264.827	146.733	212.084	142.436	226.462	133.879	171.689	122.589
6	188.513	110.312	218.62	138.5	286.772	134.418	260.695	148.396	218.868	147.062	214.61	136.187	168.079	119.196
7	164.487	106.283	256.692	144.399	298.965	140.432	260.973	148.612	207.047	152.459	215.134	133.322	183.278	138.616
8	176.755	136.599	234.934	126.401	294.957	142.436	255.066	156.623	214.159	143.281	174.235	124.024	164.12	118.79
9	170.643	136.364	220.816	142.38	282.782	138.601	250.741	134.598	183.278	130.733	159.182	125.619	169.069	126.829
10	128.342	132.414	217.171	142.507	282.868	140.432	236.767	134.598	230.85	122.736			161.738	117.36
11	150.414	130.348	218.666	136.364	274.996	130.594	238.67	120.738	203.085	130.902			179.016	122.589
12	124.477	112.317	248.865	126.337	272.845	130.409	242.854	118.468	182.971	121.981			174.73	121.137
13	124.735	112.317	224.742	132.414	266.711	130.486	208.903	110.948	190.032	135.565			140.889	118.383
14	102.587	128.358	220.597	118.383	258.884	116.379	204.545	128.733	162.545	105.219			107.991	115.286
15	82.61	118.316	192.513	122.474	240.675	118.333	200.575	112.46	170.466	124.912			134.896	121.601
16	80.838	92.268	197.758	116.327	250.668	130.733	164.744	114.936	132.732	106.755				
17			208.566	106.302	224.608	122.474	150.882	128.358						
18					214.657									

ELI-DYC-2-8-002A	Line1		Line2		Line3		Line4		Line5		Line6		Line7	
	Width	Length	Width	Length	Width	Length	Width	Length	Width	Length	Width	Length	Width	Length
1													218.618	122.047

2											200.576	128.864	203.72	118.978
3											190.549	121.731	213.001	111.144
4												150.615	203.278	105.44
5												113.011	183.924	113.272
6												118.079	168.764	114.893
7												130.212	156.83	114.893
8												125.941	209.371	120.576
9											207.145	128.285	203.247	119.334
10											193.379	144.639	188.911	115.584
11											179.772	109.374	147.992	113.68
12											167.296	103.808	172.578	106.422
13											157.059	106.543	185.482	99.061
14											157.711	95.214	181.542	119.667
15			161.668	165.211	162.523	147.748					157.573	92.241	162.294	106.976
16			140.001	93.787	142.059	122.268					143.918	98.593	150.273	112.362
17			156.83	132.964	141.869	120.884					133.629	105.647	151.49	97.966
18			137.876	103.919	129.876	127.098							145.868	99.371
19			132.964	103.919	133.311	131.858							128.365	102.576
20			134.538	98.319	136.057	127.602								
21			120.192	119.603	126.724	111.594								
22														

ELI-DYC-2-8-003A	Line1		Line2		Line3		Line4		Line5		Line6		Line7		Line8		Line9		Line10	
	Width	Length	Width	Length	Width	Length	Width	Length	Width	Length	Width	Length	Width	Length	Width	Length	Width	Length	Width	Length
1													171.638	104.766	168.682	136.319				
2													164.421	105.212	168.807	116.318	149.353	105.691	81.301	86.731
3	81.833	100.094			195.878		194.561	109.287	159.466	118.786		114.055	144.403	105.212	169.593	106.556	155.974	119.272	77.248	109.756
4	78.412	85.67			207.259	132.216	127.025	114.473	178.049	137.739		121.71	148.094	110.048	179.364	112.499	153.601	115.184	82.756	96.206
5	64.854	106.255			181.435	91.27	199.182	132.902	158.999	142.438		124.97	140.85	110.432	171.075	114.055	137.819	115.208	59.621	92.141
6	81.485	84.894			190.691	118.531	195.141	113.125	158.6	121.597		116.728	142.38	100.855	167.112	110.432	139.888	113.853	52.846	93.584
7	98.428	81.398			186.067	109.698	191.652	115.272	144.993	119.195		119.863	128.755	101.01	154.495	110.057	131.778	109.79	50.154	88.242
8	78.412	92.283			162.647	113.757	187.619	109.11	157.834	133.212		122.702	131.688	104.898	161.252	106.116	126.133	104.477	62.331	97.796
9	104.935	113.482			152.546	124.049	191.824	125.695	150.431	123.038		126.482	130.088	100.5	151.816	104.345	115.176	109.79	58.329	99.064
10	81.833	84.559			188.639	117.481	177.832	96.235	157.322	118.825		122.432	116.728	104.345	153.266	100.308	116.539	96.244	61.216	88.585
11	52.625	93.882			178.224	111.639	180.705	113.279	132.438	115.566		112.474	121.951	98.925	168.218	100.28	105.726	93.741	58.658	100.719
12	56.897	101.873			174.65	105.908	204.086	114.473	121.196	112.76		109.965	93.506	82.656	144.986	101.635	117.956	114.111	58.407	89.687
13	58.736	99.929			162.884	102.068	195.798	117.316	128.24	108.613		107.123	100.308	105.691	128.79	94.938	105.7	97.711	53.123	100.5
14	72.656	95.355			145.498	89.431	190.518	110.954	133.44	117.956		94.938	99.148	92.3	135.61	95.093	107.046	94.122	55.704	75.99
15	51.306	92.283			149.924	104.371	169.918	94.938	133.13	101.662		111.243	79.992	96.244	135.934	88.118	97.599	88.118	57.488	86.816
16					147.099	93.535	137.819	90.786	132.133	101.707		96.672	84.055	82.656	120.009	92.3	101.951	112.67	61.709	90.796
17					129.437		139.593						69.225	77.236	115.97	86.985	108.477	77.343	50.136	85.538
18															111.515	82.933	93.535			

ELI-DYC-2-8-004	Line1		Line2		Line3		Line4	
	Width	Length	Width	Length	Width	Length	Width	Length
1	199.688	95.612	215.593	83.553	195.126	93.515	225.831	107.739
2	179.041	95.612	193.254	71.252	186.997	97.642	219.514	107.72
3	174.472	99.777	221.687	65.07	211.463	79.292	219.514	103.675

4	207.35	91.551	207.4	75.228	197.19	61.007	203.286	99.777
5	203.255	93.846	199.222	85.387	219.655	73.619	203.499	87.419
6	215.478	105.688	186.986	103.735	174.838	91.663	205.288	91.483
7	207.32	103.735	184.953	93.691	182.921	111.859		
8	209.343	101.947	185.132	83.355	186.997	101.704		
9	185.232	101.643	178.96	81.324	193.126	113.981		
10	197.316	99.673	162.609	91.46	203.337	101.805		
11	195.158	109.771	176.835	85.387	201.377	93.691		
12			170.726	103.655	187.162	89.451		
13			160.615	83.355	178.856	89.52		

ELI-DYC-2-8-005	Line1		Line2		Line3		Line4		Line5	
	Width	Length	Width	Length	Width	Length	Width	Length	Width	Length
1	226.648	142.466	294.215	146.381	323.185	147.229	348.045	148.765	316.308	132.899
2	207.072	142.792	292.342	148.049	328.257	159.328	344.071	153.587	304.04	142.212
3	210.484	152.234	286.921	155.017	319.231	146.397	349.107	146.873	310.014	132.899
4	207.451	139.954	279.043	149.828	311.101	153.367	322.801	153.305	297.721	127.734
5	203.845	132.636	268.8	146.734	304.096	153.253	328.132	136.253	277.655	150.243
6	177.098	163.986	244.504	144.703	307.342	151.031	308.454	154.009	249.934	148.295
7	171.272	147.125	247.655	146.522	308.003	152.005	313.839	144.078	237.933	147.097
8	145.675	125.401	245.228	148.39	295.754	146.218	304.891	145.287	241.042	142.605
9	125.98	137.48	233.636	144.819	288.988	144.699	305.922	144.311		
10	102.314	118.191	208.865	142.41	287.326	131.835	313.377	135.82		
11	99.927	120.602	212.139	133.929	273.983	139.076				
12			199.844	135.182	266.614	139.928				
13			199.221	142.466						

ELI-DYC-2-8-007	Line1	
	Width	Length
1	317.752	105.454
2	317.477	117.359
3	361.491	93.5
4	329.674	115.919
5	339.643	117.129
6	336.962	125.663

ELI-DYC-2-8-016A	Line1		Line2		Line3		Line4		Line5	
	Width	Length	Width	Length	Width	Length	Width	Length	Width	Length
1	167.018	117.465	174.957	109.617	174.133	131.253	215.896	120.995	198.811	123.505
2	179.79	112.51	183.692	101.279	167.353	117.412	218.82	116.763	202.127	126.535
3	194.7	122.256	167.657	99.059	170.105	123.077	211.538	127.054	181.627	125.274
4	182.216	127.52	173.068	109.627	162.757	123.925	187.451	114.982	175.72	128.17
5	178.847	116.826	168.354	102.964	164.652	121.087	200.33	122.733	179.048	127.52
6	166.978	121.447	159.709	104.272	168.354	122.302	200.189	128.774	171.482	113.516
7	168.96	120.329	160.945	113.634	162.426	134.765	185.448	128.398	177.787	110.155
8	159.67	115.062	153.13	111.503	154.372	131.755	183.129	129.347	171.482	107.498
9	159.67	115.181	154.578	106.68	149.51	122.713	188.568	122.099	169.712	109.279
10	153.33	128.199	154.909	107.633	148.339	117.325	188.631	113.141	153.37	107.946

11	140.273	113.584	132.988	109.042	132.355	111.6	174.991	111.728	150.124	91.102
12	132.501	105.542	136.109	90.743	118.193	109.068	161.302	108.317	148.141	104.395
13	152.389	105.733	134.93	96.13	108.317	106.417	154.431	100.853	148.801	94.988
14	148.843	102.354	131.552	96.535	103.074	96.09	149.259	103.525	145.482	112.539
15	134.364	106.069	125.208	94.875	105.957	96.042	137.205	105.733	137.962	112.384
16	129.029	99.394	119.469	86.001	92.702	111.834	132.362	103.639		
17			108.923	83.151	97.633	106.103	138.714	104.193		
18			116.505	83.052	100.554	117.566	133.993	109.891		
19			110.223	96.528						

ELI-DYC-2-8-016B	Line1		Line2		Line3		Line4		Line5		Line6	
	Width	Length	Width	Length	Width	Length	Width	Length	Width	Length	Width	Length
1	155.372	130.752	161.366	117.771					222.494	122.178	209.897	113.465
2	152.208	119.993	161.154	122.113					234.634	111.874	185.717	125.062
3	159.654	122.335	159.142	111.688					203.117	130.179	191.433	115.885
4	163.37	113.934	144.994	110.709					177.081	115.774	173.907	113.107
5	156.467	109.813	138.443	103.433					194.749	121.916	181.674	106.658
6	132.809	97.457	144.625	101.496					198.354	136.752	188.862	118.219
7	132.632	103.324	134.692	99.515					175.665	134.438	209.694	120.472
8	137.917	101.994	135.308	97.457					169.532	124.119	205.208	123.965
9	129.423	95.845	114.551	91.574					181.826	113.934	199.031	136.424
10			117.189	111.348					186.13	114.103	177.695	111.645
11			119.676	99.279					188.738	120.117	165.493	105.552
12									174.776	107.931		
13									159.302	109.988		
14									153.975	104.964		
15									172.564	108.405		
16									151.853	104.229		

ELI-DYC-2-8-014	Line1		Line2	
	Width	Length	Width	Length
1	308.655	149.461	330.116	176.941
2	308.224	144.105	328.322	137.176
3	309.741	135.879	310.931	139.868
4	304.501	155.085	307.457	146.543
5	284.446	146.82	298.923	134.897
6	281.446	149.461	298.923	136.096
7	284.446	144.028	273.342	144.233
8	257.832	146.72		
9	242.547	133.134		

ELI-DYC-2-5-002	Line1		Line2		Line3	
	Width	Length	Width	Length	Width	Length
1	301.43	101.94	321.849	124.126	343.481	157.352
2	318.873	101.534	304.196	106.658	310.087	146.853
3	319.122	103.162	321.697	122.378	265.941	117.341
4	317.128	87.482	316.434	110.195	268.304	99.788

5			311.233	99.712	286.905	106.701

ELI-XX-001	Line1		Line2		Line3	
	Width	Length	Width	Length	Width	Length
1	265.759	154.547	303.589	147.199	317.919	140.873
2	257.847	153.952	299.424	143.051	322.56	141.221
3	252.114	152.281	312.62	138.833	329.54	146.673
4	231.175	162.326	313.252	140.504	345.967	135.254
5	263.831	142.317	301.507	142.697	352.942	146.135
6	233.449	145.514	305.03	133.395	349.213	139.324
7	216.632	140.854	287.145	139.697	345.434	143.051
8	191.465	138.684	297.716	149.454	345.412	149.454
9	208.168	147.222	286.362	128.988	324.508	137.639

ELI-XX-002	Line1	
	Width	Length
1	147.575	122.61
2	148.627	110.553
3	143.71	124.726
4	143.946	117.673
5	134.21	109.322
6	150.873	103.387
7	129.72	95.019
8	127.078	105.727

ELI-ZJB-X10-001	Line1		Line2		Line3		Line4		Line5		Line6		Line7		Line8		Line9	
	Width	Length	Width	Length	Width	Length	Width	Length	Width	Length	Width	Length	Width	Length	Width	Length	Width	Length
1	356.33	122.882	308.251	124.043	347.634	115.532	323.155	131.06	348.31	134.422	336.342	127.992	348.946	124.804	306.704	124.912	278.73	125.861
2	369.819	130.937	299.278	137.347	334.73	110.312	332.176	133.27	335.505	132.415	335.819	134.422	327.123	120.617	329.847	138.465	293.06	115.462
3	349.403	105.295	303.718	118.855	326.982	126.461	357.996	132.088	374.945	136.815	351.378	133.189	351.194	145.177	320.087	133.27	281.434	126.054
4	337.209	112.497	271.735	118.696	323.289	131.081	363.862	134.18	365.827	154.349			341.235	129.002	334.956	135.604		
5	370.374	124.804	287.659	120.055	359.571	140.789												
6	343.179	145.697			373.956	110.948												

ELI-ZJB-X10-002	Line1		Line2		Line3		Line4	
	Width	Length	Width	Length	Width	Length	Width	Length
1	257.719	86.56	227.923	101.764	271.951	99.338	324.342	121.707
2	266.407	99.688	230.461	85.344	263.118	100.445	290.31	103.051
3	307.343	86.997	246.095	82.326	291.783	81.066	260.333	110.705
4	277.787	84.218	272.591	94.988	246.949	87.414	262.166	98.603
5	280.323	92.056	313.941	80.916	290.143	107.652	263.945	107.975
6	303.14	111.319	292.431	103.461	288.32	114.829	263.204	108.213
7	307.777	117.089	269.573	114.829	300.701	111.033	308.323	89.604
8	291.305	106.407	276.689	106.407	245.694	99.703		
9	290.941	99.703	325.875	106.236	279.771	99.703		
10	355.643	98.542	282.374	95.227	311.981	105.376		
11			316.513	94.733	242.916	92.843		

12			298.887	98.971					
13			289.097	107.37					

Supplementary Data 2 Character taxon matrix in NEXUS format.

#NEXUS
BEGIN DATA;
DIMENSIONS NTAX=22 NCHAR=50;
FORMAT DATATYPE=standard GAP=- MISSING=? SYMBOLS = "0 1 2 3 4 5";
MATRIX

*Eoobolus*00110--0----0-0-0--0----0-00000--0-0000000-0010---
*Phoronis*000----0----0-0-0--0----0-00000--0-0000000-0010---
*Protomelission*111??000121010001000-1100-00000-00-000000010010000
*Dayingomelission*111??140121000001000-1100-00001001000000010010000
*Plumatella*110--4410001000-0--0-20-0-00000-00-000001020000100
*Alcyonidium*110--1400301010-0--0-20-0-00100-00-000000020100211
*Flustrellidra*110--4410301010-0--0-20-0-00100-00-000000020110210
*Arachnidium*110--1410201010-0--0-20-0-00000-10-000000020100231
*Goryunovia*111001410002000-1-1110010-00000-110010010001001011
*Corynotrypa*111001410012000-1-1110000-00000-10-000010000001011
*Hallopora*11101451121101111110-11-1010000-110000000000011211
*Orbiramus*11101451131100111100-11-100000111100000000000011210
*Profistulipora*111002501311000-1-00-1?-10000010110100100000111211
*Ceramopora*11100250131001111100-11-1111000-10-000100000101210
*Nekhorosheviella*11100250131101001-00-11-11000010110000000000001011
*Dianulites*11100250131101001-00-11-1?00000-111000000000001011
*Trepocryptopora*11101001121011101100-11-0-100?10110000000000011130
*Prophyllodictya*11101001121011111100-11-0-00011000-110000000011130
*Nematopora*11101031121000101010-1110-00011011100000000000011130
*Alwynopora*11100441011200101110-1?-0-00000-10-0000001000?1000
*Fenestrapora*111013410111010-1-10-1000-11011000-100000100011000
*Moorephylloporina*11101341011100101110-1000-00001010-000000100011000
;
END;

Supplementary Data 3 Character traits and morphological states for bryozoans.

Include 22 taxa and 50 characters used to identify phylogenetic relationships.

Gap=- Missing=? Symbols = "0 1 2 3 4 5"

No.	Character traits	States	Ref.
**1**	**Overall organization**	**0 Solitary**<br>**1 Colony**	**1**
**2**	**Attachment to substrate**	**0 Pedicle**<br>**1 Cement**	**1**
**3**	**Biomineralization**	**0 Absent**<br>**1 Present**	**2**
**4**	**Mineralogy**	**0 Calcareous**<br>**1 Apatite**	**2**
**5**	**Wall laminated**	**0 Absent**<br>**1 Present**	**3**
**6**	**Colony form**	**0 Bifoliate**<br>**1 Encrusting**<br>**2 Massive**<br>**3 Fenestrate**<br>**4 Ramose/Dendroid**	**4**
**7**	**Arrangement of frontal surface**	**0 Bilaminate**<br>**1 Trilaminate**<br>**2 Quadrate**<br>**3 Radial**<br>**4 Unilaminate**<br>**5 Multilaminate**	**5,6**
**8**	**Bifurcation**	**0 Absent**<br>**1 Present**	**5,6**
**9**	**Dimensions of growth**	**0 Two-dimensions**<br>**1 Three-dimensions**	**5,6**
**10**	**Arrangement of zooecial series**	**0 Uniserial**<br>**1 Biserial**<br>**2 Oligoserial (3-12)**<br>**3 Macroserial**	**5,6**
**11**	**Construction (proxy for flexibility)**	**0 Soft**<br>**1 Rigid**	**5,6**
**12**	**Zooid arrangement**	**0 Hexagonal or rhombic**<br>**1 Irregular**<br>**2 Linear**	**4**
**13**	**Mesotheca**	**0 Absent**<br>**1 Present**	**4**
**14**	**Zooid differentiation**	**0 Monomorphism**	**7-9**

		1 Polymorphism	
15	Endozone and exozone differentiation	0 Absent<br>1 Present	3
16	Wall thickening at boundary between endzone and exozone	0 Absent<br>1 Present	4
17	Vertical wall	0 Absent<br>1 Present	2
18	(Zooecia) cross-section in endozone	0 Polygonal<br>1 Round-sub round	4
19	(Zooecia) tangential-section	0 Polygonal<br>1 Round<br>2 Rod-like	8
20	Calcified exterior frontal wall	0 Absent<br>1 Present	2
21	Frontal wall curvature	0 Flat<br>1 Curved	8
22	Frontal wall type/<br>interior wall communication	0 Fixed-walled<br>1 Free-walled<br>2 Stolon	3,10
23	Basal wall curvature	0 Flat<br>1 Curved	4,11
24	Frontal wall (external) micro-ornamentation	0 Absent<br>1 Present	3,9
25	Maculae (monticules)	0 Absent<br>1 Present	5
26	Maculae shape	0 Irregular<br>1 Regular	4
27	Mesozooid	0 Absent<br>1 Present	4
28	Exilazooid	0 Absent<br>1 Present	4,10
29	Kenozooid	0 Absent<br>1 Present	3
30	Exilazooidal skeleton (stereo)	0 Absent<br>1 Present	4
31	Style	0 Absent<br>1 Present	3
32	Style size modality	0 Unimodal<br>1 Polymodal	8
33	Length of autozooid	0 Short<br>1 Long (> 2 zooid chamber)	8
34	Zooid diaphragm/septa	0 Absent<br>1 Present	4

**35**	**Zoid diaphragm spacing**	**0 Close (< 1 living chamber)**<br>**1 Wide (> 1 living chamber)**	**4**
**36**	**Vesicular tissue**	**0 Absent**<br>**1 Present**	**4**
**37**	**Hemiphragm**	**0 Absent**<br>**1 Present**	**4**
**38**	**Medial zooid**	**0 Absent**<br>**1 Present**	**4**
**39**	**Lunaria**	**0 Absent**<br>**1 Present**	**8**
**40**	**Capitulum**	**0 Absent**<br>**1 Present**	**9**
**41**	**Cauda**	**0 Absent**<br>**1 Present**	**9**
**42**	**Median keel**	**0 Absent**<br>**1 Present**	**12**
**43**	**Zooid shape**	**0 Tube**<br>**1 Box**<br>**2 Vase**	**2,3**
**44**	**Pseudopores**	**0 Absent**<br>**1 Present**	**3**
**45**	**Pore chamber**	**0 Absent**<br>**1 Present**	**3**
**46**	**Holdfast**	**0 Absent**<br>**1 Present**	**1**
**47**	**Granular wall**	**0 Absent**<br>**1 Present**	**4**
**48**	**Budding geometry**	**0 Simple linear**<br>**1 Compound linear**<br>**2 Non-linear**	**13**
**49**	**Budding loci**	**0 Distal**<br>**1 Lateral+Distal**<br>**2 Frontal+Distal**<br>**3 Spiral**	**9,13**
**50**	**Regularity of budding loci**	**0 Regular**<br>**1 Irregular (more than one sites)**	**9,13**

References

[1] Boardman, R. S. et al. Treatise on invertebrate paleontology. Part G. Bryozoa. vol. 1 (Geological Society of America, Boulder, CO & University of Kansas Press, 1983).

[2] Taylor, P. D., Lombardi, C. & Cocito, S. Biomineralization in bryozoans: present, past and future: Bryozoan biomineralization. Biol. Rev. 90, 1118–1150 (2015).

[3] Cuffey, R. J. & Blake, D. B. Cladistic analysis of the phyllum Bryozoa. Bull. Société Sci. Nat. Ouest Fr. 97–108 (1991).

[4] Ma, J., Taylor, P. D., Xia, F. & Zhan, R. The oldest known bryozoan: Prophyllodictya (Cryptostomata) from the lower Tremadocian (Lower Ordovician) of Liujiachang, south-western Hubei, central China. Palaeontology 58, 925–934 (2015).

[5] Hageman, S. J., Bock, P. E., Bone, Y. & Mcgowran, B. Bryozoan growth habits: Classification and analysis. J. Paleontol. 72, 418–436 (1998).

[6] Hageman, S. J. Complexity generated by iteration of hierarchical modules in Bryozoa. Integr. Comp. Biol. 43, 87–98 (2003).

[7] McKinney, F. K. & Jackson, J. B. C. Bryozoan Evolution. (University of Chicago Press, 1989).

[8] Ma, J.-Y., Buttler, C. J. & Taylor, P. D. Cladistic analysis of the 'trepostome' Suborder Esthonioporina and the systematics of Palaeozoic bryozoans. Bryozoan Stud. 94, 153–161 (2014).

[9] Todd, J. A. The central role of ctenostomes in bryozoan phylogeny. Proc. 11th Int. Bryozool. Assoc. Conf. 104, 104–135 (2000).

[10] Taylor, P. D. Bryozoan Paleobiology. (John Wiley & Sons, 2020).

[11] Ernst, A. & Carrera, M. Upper Ordovician (Sandbian) bryozoan fauna from argentine precordillera. J. Paleontol. 86, 721–752 (2012).

[12] Ernst, A. Fenestrapora (Fenestrata, Bryozoa) from the Middle Devonian of Germany. Paläontol. Z. 90, 19–32 (2016).

[13] Lidgard, S. Zooid and colony growth in encrusting cheilostome bryozoans. Palaeontology 28, 255–291 (1985).

[14] Schwaha, T. (Ed.). Phylum Bryozoa. Walter de Gruyter GmbH & Co KG (2020).

Supplementary Data 4 Character trait dataset-matrix

	Matrix																						
	**No.**	**1**	**2**	**3**	**4**	**5**	**6**	**7**	**8**	**9**	**10**	**11**	**12**	**13**	**14**	**15**	**16**	**17**	**18**	**19**	**20**	**21**	**22**
Outgroup	*Eoobolus*	0	0	1	1	0	-	-	0	-	-	-	-	0	-	0	-	0	-	-	0	-	-
	Phoronis	0	0	0	-	-	-	-	0	-	-	-	-	0	-	0	-	0	-	-	0	-	-
Materials in this study	*Protomelission*	1	1	1	?	?	0	0	0	1	2	1	0	1	0	0	0	1	0	0	0	-	1
	Dayingomelission	1	1	1	?	?	1	4	0	1	2	1	0	0	0	0	0	1	0	0	0	-	1
Phylactolaemata	*Plumatella*	1	1	0	-	-	4	4	1	0	0	0	1	0	0	0	-	0	-	-	0	-	2
Gymnolaemata	*Alcyonidium*	1	1	0	-	-	1	4	0	0	3	0	1	0	1	0	-	0	-	-	0	-	2
	Flustrellidra	1	1	0	-	-	4	4	1	0	3	0	1	0	1	0	-	0	-	-	0	-	2
	Arachnidium	1	1	0	-	-	1	4	1	0	2	0	1	0	1	0	-	0	-	-	0	-	2
Cyclostomata	*Goryunovia*	1	1	1	0	0	1	4	1	0	0	0	2	0	0	0	-	1	-	1	1	1	0
	Corynotrypa	1	1	1	0	0	1	4	1	0	0	1	2	0	0	0	-	1	-	1	1	1	0
Trepostomata	*Hallopora*	1	1	1	0	1	4	5	1	1	2	1	1	0	1	1	1	1	1	1	0	-	1
	Orbiramus	1	1	1	0	1	4	5	1	1	3	1	1	0	0	1	1	1	1	0	0	-	1
Cystoporata	*Profistulipora*	1	1	1	0	0	2	5	0	1	3	1	1	0	0	0	-	1	-	0	0	-	1
	Ceramopora	1	1	1	0	0	2	5	0	1	3	1	0	0	1	1	1	1	1	0	0	-	1
Esthonioporata	*Nekhorosheviella*	1	1	1	0	0	2	5	0	1	3	1	1	0	1	0	0	1	-	0	0	-	1
	Dianulites	1	1	1	0	0	2	5	0	1	3	1	1	0	1	0	0	1	-	0	0	-	1
Cryptostomata	*Trepocryptopora*	1	1	1	0	1	0	0	1	1	2	1	0	1	1	1	0	1	1	0	0	-	1
	Prophyllodictya	1	1	1	0	1	0	0	1	1	2	1	0	1	1	1	1	1	1	0	0	-	1
	Nematopora	1	1	1	0	1	0	3	1	1	2	1	0	0	0	1	0	1	0	1	0	-	1
Fenestrata	*Alwynopora*	1	1	1	0	0	4	4	1	0	1	1	2	0	0	1	0	1	1	1	0	-	1
	Fenestrapora	1	1	1	0	1	3	4	1	0	1	1	1	0	1	0	-	1	-	1	0	-	1
	Moorephylloporina	1	1	1	0	1	3	4	1	0	1	1	1	0	0	1	0	1	1	1	0	-	1

Character traits																											
**23**	**24**	**25**	**26**	**27**	**28**	**29**	**30**	**31**	**32**	**33**	**34**	**35**	**36**	**37**	**38**	**39**	**40**	**41**	**42**	**43**	**44**	**45**	**46**	**47**	**48**	**49**	**50**
-	-	0	-	0	0	0	0	0	-	-	0	-	0	0	0	0	0	0	0	-	0	0	1	0	-	-	-
-	-	0	-	0	0	0	0	0	-	-	0	-	0	0	0	0	0	0	0	-	0	0	1	0	-	-	-
1	0	0	-	0	0	0	0	0	-	0	0	-	0	0	0	0	0	0	0	1	0	0	1	0	0	0	0
1	0	0	-	0	0	0	0	1	0	0	1	0	0	0	0	0	0	0	0	1	0	0	1	0	0	0	0
0	-	0	-	0	0	0	0	0	-	0	0	-	0	0	0	0	0	1	0	2	0	0	0	0	1	0	0
0	-	0	-	0	0	1	0	0	-	0	0	-	0	0	0	0	0	0	0	2	0	1	0	0	2	1	1
0	-	0	-	0	0	1	0	0	-	0	0	-	0	0	0	0	0	0	0	2	0	1	1	0	2	1	0
0	-	0	-	0	0	0	0	0	-	1	0	-	0	0	0	0	0	0	0	2	0	1	0	0	2	3	1
0	1	0	-	0	0	0	0	0	-	1	1	0	0	1	0	0	1	0	0	0	1	0	0	1	0	1	1
0	0	0	-	0	0	0	0	0	-	1	0	-	0	0	0	0	1	0	0	0	0	0	0	1	0	1	1
1	-	1	0	1	0	0	0	0	-	1	1	0	0	0	0	0	0	0	0	0	0	0	1	1	2	1	1
1	-	1	0	0	0	0	0	1	1	1	1	0	0	0	0	0	0	0	0	0	0	0	1	1	2	1	0
?	-	1	0	0	0	0	0	1	0	1	1	0	1	0	0	1	0	0	0	0	0	1	1	1	2	1	1
1	-	1	1	1	1	0	0	0	-	1	0	-	0	0	0	1	0	0	0	0	0	1	0	1	2	1	0
1	-	1	1	0	0	0	0	1	0	1	1	0	0	0	0	0	0	0	0	0	0	0	0	1	0	1	1
1	-	1	?	0	0	0	0	0	-	1	1	1	0	0	0	0	0	0	0	0	0	0	0	1	0	1	1
1	-	0	-	1	0	0	?	1	0	1	1	0	0	0	0	0	0	0	0	0	0	0	1	1	1	3	0
1	-	0	-	0	0	0	1	1	0	0	0	-	1	1	0	0	0	0	0	0	0	0	1	1	1	3	0
1	1	0	-	0	0	0	1	1	0	1	1	1	0	0	0	0	0	0	0	0	0	0	1	1	1	3	0
?	-	0	-	0	0	0	0	0	-	1	0	-	0	0	0	0	0	0	1	0	0	0	?	1	0	0	0
0	0	0	-	1	1	0	1	1	0	0	0	-	1	0	0	0	0	0	1	0	0	0	1	1	0	0	0
0	0	0	-	0	0	0	0	1	0	1	0	-	0	0	0	0	0	0	1	0	0	0	1	1	0	0	0
