# Supplementary material for: High-fidelity modular skeletons authenticate a Cambrian origin for Bryozoa
Source: Nature. 2026 Jun 3;656(8126):123–8. doi: 10.1038/s41586-026-10590-9 (PMC13441974; doi:10.1038/s41586-026-10590-9)
Supplement: Supplementary file 2 [file 41586_2026_10590_MOESM2_ESM.pdf]

nature portfolio

Corresponding author(s): Zhifei Zhang

Last updated by author(s): Apr 18, 2026

Reporting Summary

Nature Portfolio wishes to improve the reproducibility of the work that we publish. This form provides structure for consistency and transparency in reporting. For further information on Nature Portfolio policies, see our Editorial Policies and the Editorial Policy Checklist.

Statistics

For all statistical analyses, confirm that the following items are present in the figure legend, table legend, main text, or Methods section.

n/a	Confirmed	
☐	☒	The exact sample size (*n*) for each experimental group/condition, given as a discrete number and unit of measurement
☐	☒	A statement on whether measurements were taken from distinct samples or whether the same sample was measured repeatedly
☒	☐	The statistical test(s) used AND whether they are one- or two-sided<br>*Only common tests should be described solely by name; describe more complex techniques in the Methods section.*
☒	☐	A description of all covariates tested
☒	☐	A description of any assumptions or corrections, such as tests of normality and adjustment for multiple comparisons
☒	☐	A full description of the statistical parameters including central tendency (e.g. means) or other basic estimates (e.g. regression coefficient) AND variation (e.g. standard deviation) or associated estimates of uncertainty (e.g. confidence intervals)
☒	☐	For null hypothesis testing, the test statistic (e.g. *F*, *t*, *r*) with confidence intervals, effect sizes, degrees of freedom and *P* value noted<br>*Give P values as exact values whenever suitable.*
☐	☒	For Bayesian analysis, information on the choice of priors and Markov chain Monte Carlo settings
☒	☐	For hierarchical and complex designs, identification of the appropriate level for tests and full reporting of outcomes
☒	☐	Estimates of effect sizes (e.g. Cohen's *d*, Pearson's *r*), indicating how they were calculated

Our web collection on statistics for biologists contains articles on many of the points above.

Software and code

Policy information about availability of computer code

Data collection	ImageJ 1.54f for size measurement.
Data analysis	ORS Dragonfly, v. 2022.2.0.1367 for uCT reconstruction, visualisation and segmentation. MrBayes v.3.2.7 (open source) for phylogenetic analyses. The settings needed to replicate these analyses are provided in the paper.

For manuscripts utilizing custom algorithms or software that are central to the research but not yet described in published literature, software must be made available to editors and reviewers. We strongly encourage code deposition in a community repository (e.g. GitHub). See the Nature Portfolio guidelines for submitting code & software for further information.

Data

Policy information about availability of data

All manuscripts must include a data availability statement. This statement should provide the following information, where applicable:

- Accession codes, unique identifiers, or web links for publicly available datasets
- A description of any restrictions on data availability
- For clinical datasets or third party data, please ensure that the statement adheres to our policy

All data analysed in this paper, including the phylogenetic datasets, are available as part of the Article, Extended Data Figs. 1–7, Extended Data 1, Extended Data Table 1, Supplementary Information.

Research involving human participants, their data, or biological material

Policy information about studies with human participants or human data. See also policy information about sex, gender (identity/presentation), and sexual orientation and race, ethnicity and racism.

Reporting on sex and gender	No human participants, human data, or human biological material were involved in this study.
Reporting on race, ethnicity, or other socially relevant groupings	The study did not involve human participants or human data; therefore, no socially relevant categorization variables were applicable.
Population characteristics	The study did not involve human research participants; no population characteristics are applicable.
Recruitment	No participants were recruited, as the study did not involve human participants.
Ethics oversight	No ethics approval was required, because this study did not involve human participants, their data, or biological material.

Note that full information on the approval of the study protocol must also be provided in the manuscript.

Field-specific reporting

Please select the one below that is the best fit for your research. If you are not sure, read the appropriate sections before making your selection.

☐ Life sciences ☐ Behavioural & social sciences ☒ Ecological, evolutionary & environmental sciences

For a reference copy of the document with all sections, see nature.com/documents/nr-reporting-summary-flat.pdf

Ecological, evolutionary & environmental sciences study design

All studies must disclose on these points even when the disclosure is negative.

Study description	This is a palaeontological and taxonomic study of early Cambrian materials from South China, involving collection, preparation, high-resolution imaging, and phylogenetic analysis. Exceptionally preserved soft tissues definitively place these taxa as crown-group Stenolaemata, confirming a Cambrian origin for bryozoans.
Research sample	The specimens used represent all currently known specimens of P. gatehousei and Dayingomelission hexaclitia gen. et sp. nov. (including the holotype) and illustrate all the key taxonomic features of these two species. This evidence are more than adequate to establish that P. gatehousei and Dayingomelission hexaclitia gen. et sp. nov. are crown group stenolaemates.
Sampling strategy	We utilise all currently known specimens of P. gatehousei and Dayingomelission hexaclitia gen. et sp. nov.(including the holotype). These specimens provide all the necessary information to establish the evolutionary affinities of these two species.
Data collection	Zhifei Zhang led the fossil excavation at the Dayingcun, Zhujiaba, Yangjiagou sections in 2020—2025, and Baopeng Song, Yanlong Chen and Mei Luo recovered 38 specimens (ELI DYCX 5-001–002; ELI DYCX 8-001–016; ELI DYCX 9-001; ELI ZJBX 3-001; ELI ZJBX 10-001–002) from acid macerated residues in 2021-2025. SEM, BSE and EDS images were collected using a Zeiss Supra 35 VP field emission, Fei Quanta 450-FEG and Phenom XL G2. µCT projections were collected using a Zeiss Xradia CrystalCT. Ultra-thin foils, HAADF images, SAED images and STEM-EDXS mappings were performed using Zeiss Auriga Compact dual beam FIB-SEM instrument equipped with an Omniprobe 200 micromanipulator, JEM 2100HR TEM and JEM F200. Measurements were performed on µCT and SEM images.
Timing and spatial scale	Collection of the specimens of P. gatehousei and Dayingomelission hexaclitia gen. et sp. nov. from the Dayingcun section took place in 2020—2025. Collection of the specimens from the Zhujiaba and Yangjiagou sections took place in 2022-2023. Collection of the specimens from the Donggouwan section took place in 2025. The material collected, which represents all of the material of P. gatehousei and Dayingomelission hexaclitia gen. et sp. nov. currently known, is more than adequate to establish the evolutionary affinities of P. gatehousei and Dayingomelission hexaclitia gen. et sp. nov.
Data exclusions	No data was excluded.
Reproducibility	This is a palaeontological study that utilises fossils that form part of the evolutionary record of this planet. They cannot be replicated or duplicated. We provide ample information in our paper for anyone to resample the localities that are the sources of these specimens and to repeat the methods of analysis we use.
Randomization	No randomization was used.
Blinding	This is a palaeontological study of all known material of two genera. Blinding is inapplicable and irrelevant.

Did the study involve field work? ☒ Yes ☐ No

Field work, collection and transport

Field conditions	Lower Cambrian rocks were well-exposed and fossiliferous limestones were excavated manually in the field. The Xiannüdong Formation is exposed in an area with a mild and humid climate with an average temperature of 14°C and an average amount of rainfall of 1323 mm per year.
Location	Donggouwan Section, Hanzhong, China: 32.895105E, 107.035052N<br>Dayingcun Section, Hanzhong, China: 32.482404E, 107.231450N<br>Zhujiaba Section, Hanzhong, China: 32.495237E, 107.171065N<br>Yangjiagou Section, Hanzhong, China: 33.003197E, 107.886931N
Access & import/export	All fossil specimens in this study were collected by the field group led by Northwest University in compliance with all local, national and international laws. All collecting permissions were obtained before the collecting started. Any collecting in private land also obtained permissions from the land holder. No permits were required to collect any of the samples included in our study.
Disturbance	All sampling was collected by hand with minimal disturbance of the surrounding environment.

Reporting for specific materials, systems and methods

We require information from authors about some types of materials, experimental systems and methods used in many studies. Here, indicate whether each material, system or method listed is relevant to your study. If you are not sure if a list item applies to your research, read the appropriate section before selecting a response.

Materials & experimental systems

n/a	Involved in the study
☒	☐ Antibodies
☒	☐ Eukaryotic cell lines
☐	☒ Palaeontology and archaeology
☒	☐ Animals and other organisms
☒	☐ Clinical data
☒	☐ Dual use research of concern
☒	☐ Plants

Methods

n/a	Involved in the study
☒	☐ ChIP-seq
☒	☐ Flow cytometry
☒	☐ MRI-based neuroimaging

Palaeontology and Archaeology

Specimen provenance	All fossils were collected from bioclastic and reef limestone outcrops of the Xiannüdong Formation, Hanzhong City, China.<br>No permits were required to collect these specimens.
Specimen deposition	All specimens are deposited at Northwest University.
Dating methods	no new dates are provided in the paper.

☐ Tick this box to confirm that the raw and calibrated dates are available in the paper or in Supplementary Information.

Ethics oversight	No ethics permissions were required to undertake our study.

Note that full information on the approval of the study protocol must also be provided in the manuscript.

Plants

Seed stocks	This study did not involve plants or plant material; all materials are fossil specimens.
Novel plant genotypes	No novel plant genotypes were generated or used, as the study is palaeontological and based entirely on fossil material.
Authentication	Authentication procedures are not relevant to this study.
