# Supplementary material for: High-fidelity modular skeletons authenticate a Cambrian origin for Bryozoa
Source: Nature. 2026 Jun 3;656(8126):123–8. doi: 10.1038/s41586-026-10590-9 (PMC13441974; doi:10.1038/s41586-026-10590-9)
Supplement: Supplementary file 3 [file 41586_2026_10590_MOESM3_ESM.pdf]

nature portfolio

Peer Review File

High-fidelity modular skeletons authenticate a Cambrian origin for Bryozoa

Corresponding Author: Professor Zhifei Zhang

Any redactions in this file are there to maintain patient confidentiality, the confidentiality of unpublished data, or to remove third-party material.

This file contains all reviewer reports in order by version, followed by all author rebuttals in order by version.

Version 0:

Reviewer comments:

Referee #1

(Remarks to the Author)
Since 1990's, the Cambrian Explosion has been a hot research spot, and most extant metazoan phyla had earliest fossil records back to this radiation event. But the age of the earliest bryozoan fossil record has been an enigma, and reports of its Cambrian fossils were more or less in debate. A convinced recovery of early Cambrian bryozoan fossils consistent with molecular clock analyses has been an enthusiastic expectation and hard efforts. Congratulations! This manuscript reports convincing bryozoan fossils from the lower Cambrian of South China!
Two well preserved species of bryozoan fossils through diagenetic phosphatization were recovered from the Xiannudong Formation (Cambrian Stage 3) in southern Shaanxi through acetic-acid maceration of limestone samples. Through advanced multiple advanced research methods, the fossils were well extensively studied. The phosphatized specimens are modular with preservation of many anatomical structures, e.g., membranous sacs, styles, annular muscles, and ring septa. All these features show that the two taxa belong to bryozoans, and phylogenetic analysis indicates them being crown group stenolaemates, confirming an early Cambrian origin of bryozoans, consistent with other animal phyla. This work is a very important contribution to understanding of the origin and early evolution of bryozoans, and to studying the Cambrian radiation.
It is suitable for publishing this manuscript in Nature. The manuscript is well written and organized. But there are some comment for the current version of this MS. For detailed comments, please see 'Comments to authors'.

Comments to authors
Title: may add the word 'early', early Cambrian origin!
Line 40: The origin of the colonial lophotrochozoan phylum Bryozoa
Line 41: a diverse fossil fauna---diverse fossil record?
Line 87: All zooids---All zooids of D. hexaclitia or of both taxa?
Line 89: an average width of 208.4 μm and a length of 121.7 μm. But how to define the width and length?
Lines 91-92: 'to the median lamina (mesotheca) or basal exterior wall (Fig. 2d, Extended Data Fig. 6a–c, f)'--- to the median lamina (mesotheca) (Data Fig. 6a–c, f) or basal exterior wall (Fig. 2d).
In Fig.1h: there are membranous sacs, but no vertical skeletal walls. Why were they lost during diagenesis?
Line 105: sac—sacs.
Line 121: muscles. (Fig. 1i----muscles (Fig. 1i
Line 122: The sacs---The sac membranes
Line 138: Front side --- Oblique front side
Line 143: three?
Line 147: Hexagonal cystids with styles---Oblique view, showing hexagonal cystids with styles
Line 175: soft-part --- soft-tissue? May be consistent within the MS.
Line 185: 'the first of two independent biomineralization events in the Bryozoa'. Can this be briefly introduced?
Lines 351-352: 'The phosphatized specimens were collected manually using acid maceration in 5–10% acetic acid', this sentence does not well introduce clearly how to get the specimens. Actually, you may re-organize the paragraph 'Material and occurrences' for a clear knowledge!
Line 355: Cambrian Series 2, Stage 3---Cambrian Stage 3

Lines 355-361: samples—specimens.
Line 502: arrows---arrow

Extended Data Figure 1C: what does it mean for the 'stratigraphic horizon' numbers? If they represent 'beds or layers', then the numbers should be put between the line bars. And the Xiaoyangba locality is also indicated in Figure 1b, but no specimens studied in this MS are from this locality.
Extended Data Fig. 6: the outline differences of b and c both with a same scale should be briefly introduced, especially c with a same colour.

'P. gatehousei, oligoserial colony form, tapering distally'. Is this form quite usual for bryozoans?
Orifice and aperture both being used. What is the difference? While in Extended Data Fig. 2 being orifice, it being aperture in Fig. 1J,K and in Diagnosis of new taxon.
Why vertical skeletal walls of adjacent zooids form a double wall (Extended Data Fig. 6f), while looking like a solid wall in Extended Data Fig. 3b?

Systematic Palaeontology: Why do not put the new taxon under the Class Stenolaemata if considering it as a crown group stenolaemate?
"Diagnosis—As for genus diagnosis by monotypy." This line should be under the new species name and above Description.
Within the Description paragraph, figure information may be cited for checking the features.
For the new species, a holotype should be noticed.
Both taxa are with skeletal walls. How about the wall original composition?

Referee #2

(Remarks to the Author)
Song et al. present compelling evidence supporting the Cambrian origin of the Bryozoa. They found phosphatized fossils of two bryozoan taxa from an early Cambrian formation of China. Their results are original and of great significance, because two studies (Yang et al., 2023, Nature 615: 468–471; Xiang et al., 2024, Geology 52: 130–134) published after their original finding (Zhang et al., 2021, Nature 599: 251–255) casted doubts about the bryozoan nature of this fossil.

They based their conclusions on a variety of techniques, such as Scanning Electron Microscopy, focused ion beam-Transmission Electron Microscopy, X-ray computed tomography and STEM-EDXS mapping. Phylogenetic inferences were based on Bayesian Analysis of a matrix including Protomelission and Dayingomelission, 18 bryozoan genera, two outgroups and 50 characters.

Their conclusions are robust and reliable, since they provide images of soft tissues (e.g., membranous sacs) preserved in-situ within modular structures and typical bryozoan structures such as styles. The Abstract and the Introduction are complete and appropriate, introducing the reader to fundamental studies on the subject.

The Reference list is exhaustive and up-to-date, although I found some minor problems that should be corrected before acceptance: (1) Delete the comma after the journal name on line 264. (2) Replace the colon with a period on line 266. (3) Marcusodictyon instead of Marcusodictyan on line 273, with italics. (4) Incomplete title of articles on lines 269 ("Taxonomic notes on genera of fossil and recent Bryozoa") and 276-277 ("The oldest bryozoans: new evidence from the Late Tremadocian (Early Ordovician) of East Yangtze Gorges in China)". (5) Incomplete journal name on line 245. (6) Begin with a capital letter after the author's name on line 257. (7) On line 292, the author's initials are M.A.S and in "The Cambrian Explosion:" each word begins with a capital letter. (8) The family name of the second author on line 294 is Wyse Jackson. (9) DOIs should be added whenever possible. (10) Abbreviate journal names on lines 266–267, 268, 269–270, 293, 313, 315–316, 318, 332–333, 336 and 339.

In my opinion, this study by Song et al. provides sound and reliable evidences on the Cambrian origin of Bryozoa and demonstrates an early diversification in this phylum due to the discovery of Dayingomelission. It will have a major impact on researchers aiming to elucidate the origin of invertebrate animal phyla, particularly the Lophotrochozoa.

Juan López-Gappa
Débora Iturra

Referee #3

(Remarks to the Author)
The manuscript describes a new Cambrian bryozoan taxon, and provides further information on a previously known species. The fossils are exceptionally well preserved and features of the skeleton make it clear that the material belongs to the Phylum Bryozoa and the conclusions are justified. This also resolves the contention by some authors that the species described by Zhang 2021 was erroneously assigned to bryozoans. The presence of two distinct species, with differing morphotypes. in the Cambrian also suggests a common ancestor in the early Cambrian or even Ediacaran, and this interpretation is justified. However the text is inconsistent with respect to wall mineralogy (see below for details). An interpretation is made that these biomineralized walls represent an independent mineralisation event and the authors suggest a common ancestor earlier. This suggestion of an earlier ancestor, which is justified based on two taxa with different

bryozoan morphotypes, does not hinge on biomineralization. The significance of the manuscript does not depend on being able to confirm biomineralization during life, but the authors interpretation does need to be clarified.
The methodology is appropriate and the figure presentation is of good quality (noted in the annotated pdf some figure captions need species presented defined), and the references are appropriate. The abstract is clear and appropriate to the content of the manuscript
This is a significant contribution and is worthy of publication

ADDITIONAL COMMENTS (see also the annotated pdf)
Wall mineralogy
Be clear about whether the fossils were phosphatised after death as a diagenetic process, or do you mean to say they had a phosphatic skeleton during life stages. I think you mean the former (and this was stated in the Zhang et al original description of P. gatehousei) but in places this is not clear in your manuscript. I have tried to highlight these in the annotated pdf.
The terminology in the results for both taxa refer to phosphatisation or phosphatised fossil/walls/structures – ie the phosphatisation occurred after death of the bryozoan. However in the discussion at line 183-186 it is stated that this is the first evidence of biomineralized stenolaemates, which indicates you are interpreting that they were biomineralized in life. This conflicts with the terminology given in the results.

Phosphatised wall = the wall is replaced not original mineralogy
Phosphatic wall = the wall was made of phosphate by the organism, or replaced.

The elemental maps in Extended Data Fig 2 only show that phosphate and silica are present, they do not confirm when this happened (during life or after death). So the intention of the authors needs to be made clear in the text.
What is the evidence that these fossils were made of phosphate during life? The diagenetic replacement process could result in perfect replication of an original organic wall, so if you think these were mineralised during life a case needs to be made for that. This is not to suggest that Cambrian bryozoans were not phosphatic walled, but evidence needs to be supplied or an argument made. To my knowledge the lingulids are still considered the first brachiopods, and they were phosphatic (and still are), so that bryozoans may have first been phosphatic is not out of the question. But an argument needs to be made for this.

Systematic Palaeontology – no holotype is defined in the description and the measurement tables do not have units, or identification of what structure is being measured, and what each line refers to. This must be corrected. The systematic description of D. hexaclitia should be in the extended data section.

Catherine Reid

Referee #4

(Remarks to the Author)
I co-reviewed this manuscript with one of the reviewers who provided the listed reports.

Version 1:

Reviewer comments:

Referee #1

(Remarks to the Author)
In this revised MS, the authors answered all our quesions and accepted most of our reviewers' suggestions. Great work. I am happy with their work!

I nearly have no more suggestions, but for Line 198: 'early Cambrian' may be 'early Terreneuvian' or 'early Cambrian (Terreneuvian)' since the described fossils are also described as the early Cambrian in the MS.

Referee #2

(Remarks to the Author)
The authors have addressed all the requested changes. The manuscript may now be published as it is.

Referee #3

(Remarks to the Author)
Dear Zhifei Zhang,

Thank-you for your response to my comments regarding wall mineralogy. It is now clear from the text that the fossils were phosphastised after death and that the composition of the skeleton in live remains unknown. Thank-you for moving the systematic palaeontology to the extended dxata section where it will remain a part of the archived article. These changes address my key concerns on the original manuscript and I support publication in its current form.

Regards
Catherine Reid

Referee #4

(Remarks to the Author)
I co-reviewed this manuscript with one of the reviewers who provided the listed reports.

Open Access This Peer Review File is licensed under a Creative Commons Attribution 4.0 International License, which permits use, sharing, adaptation, distribution and reproduction in any medium or format, as long as you give appropriate credit to the original author(s) and the source, provide a link to the Creative Commons license, and indicate if changes were made.
In cases where reviewers are anonymous, credit should be given to 'Anonymous Referee' and the source.
The images or other third party material in this Peer Review File are included in the article's Creative Commons license, unless indicated otherwise in a credit line to the material. If material is not included in the article's Creative Commons license and your intended use is not permitted by statutory regulation or exceeds the permitted use, you will need to obtain permission directly from the copyright holder.
To view a copy of this license, visit https://creativecommons.org/licenses/by/4.0/

Response to Reviewers' Comments

Manuscript Title: High-fidelity modular skeletons authenticate a Cambrian origin for Bryozoa

Manuscript ID: 2026-02-04304

To the Editor,

Please find attached a revised version of our manuscript "High-fidelity modular skeletons authenticate a Cambrian origin for Bryozoa", which we are resubmitting for publication in *Nature*.

We greatly appreciate the comments and suggestions provided by the four referees. We have worked assiduously to incorporate their requested changes and we believe their suggestions have enhanced the overall quality of our manuscript.

Below we provide a point-by-point response describing how we have addressed each of the referee's comments. The original comments are listed in bold and our reply, along with a description of changes we have made to the manuscript text, follows immediately after. We also have included the references cited in our responses to the referees at the end.

We thank you for taking the time to consider our manuscript for publication in *Nature* and we hope that with the changes we have made to our paper it is now ready for acceptance. If you have any further questions about our revised manuscript, please do not hesitate to get in contact. Thank you very much for your time and consideration.

Your Sincerely,

Zhifei Zhang
(on behalf of all co-authors)
Department of Geology, Northwest University, Xi'an 710069, China
elizf@nwu.edu.cn

Referee #1 (Remarks to the Author):

Since 1990's, the Cambrian Explosion has been a hot research spot, and most extant metazoan phyla had earliest fossil records back to this radiation event. But the age of the earliest bryozoan fossil record has been an enigma, and reports of its Cambrian fossils were more or less in debate. A convinced recovery of early Cambrian bryozoan fossils consistent with molecular clock analyses has been an enthusiastic expectation and hard efforts. Congratulations! This manuscript reports convincing bryozoan fossils from the lower Cambrian of South China!

Two well preserved species of bryozoan fossils through diagenetic phosphatization were recovered from the Xiannudong Formation (Cambrian Stage 3) in southern Shaanxi through acetic-acid maceration of limestone samples. Through advanced multiple advanced research methods, the fossils were well extensively studied. The phosphatized specimens are modular with preservation of many anatomical structures, e.g., membranous sacs, styles, annular muscles, and ring septa. All these features show that the two taxa belong to bryozoans, and phylogenetic analysis indicates them being crown group stenolaemates, confirming an early Cambrian origin of bryozoans, consistent with other animal phyla. This work is a very important contribution to understanding of the origin and early evolution of bryozoans, and to studying the Cambrian radiation.

It is suitable for publishing this manuscript in Nature. The manuscript is well written and organized.

Response: We thank Referee #1 for your interest in our research and your strong recommendation for publication in *Nature*.

But there are some comment for the current version of this MS. For detailed comments, please see 'Comments to authors'.

Response: We appreciate the time Referee #1 has taken to thoroughly check our manuscript and the suggested revisions provided. We have taken all of these suggestions on board and have made changes to the text accordingly.

Comments to authors

Title: may add the word 'early', early Cambrian origin!

Response: We agree that including "early" in the title would indeed make the temporal context more precise. However, due to *Nature*'s strict character limit for titles (maximum 75 characters including spaces), we are unable to incorporate this addition without exceeding the limit (74 characters now). We have therefore retained the original title while ensuring that the early Cambrian age of these fossils is clearly emphasized in both the abstract and the main text. (Line 29, 61, 187, 195, 239)

Line 40: The origin of the colonial lophotrochozoan phylum Bryozoa

Response: We have revised the sentence accordingly: "The origin of the colonial lophotrochozoan phylum Bryozoa (has often been considered an evolutionary enigma...)". (Line 41)

Line 41: a diverse fossil fauna---diverse fossil record?

Response: Corrected and revised as suggested. (Line 42)

Line 87: All zooids---All zooids of *D. hexaclitia* or of both taxa?

Response: "All zooids" should refer to both taxa. We have revised the sentence to make this clear. (Line 88)

Line 89: an average width of 208.4 µm and a length of 121.7 µm. But how to define the width and length?

Response: To address this ambiguity, in the revised measurement tables we now make clear that length refers to the longitudinal axis of zooids (parallel to the growth axis), while width refers to the transverse axis measured perpendicular to the growth direction. A fossil image illustrating these measurements has been added to Extended Data Fig. 4. The tables have also been revised to include clear units (μm) and explicit identification of all measured structures. (Line 509–516, Extended Data Fig. 4, Supplementary Data 1 Measurements of *Protomelission gatehousei* and *Dayingomelission hexaclitia* gen. et sp. nov.)

Lines 91-92: 'to the median lamina (mesotheca) or basal exterior wall (Fig. 2d, Extended Data Fig. 6a–c, f)'--- to the median lamina (mesotheca) (Data Fig. 6a–c, f) or basal exterior wall (Fig. 2d).
Response: Corrected and revised as suggested. (Line 93–94)

In Fig.1h: there are membranous sacs, but no vertical skeletal walls. Why were they lost during diagenesis?
Response: Fossil preservation during diagenesis is an inherently complex process, particularly in early Cambrian phosphatized fossils. Preservation bias arises from multiple factors, including variation in decay rates of different anatomical parts and differences in their capacity to incorporate relevant chemical elements from seawater. During early Cambrian phosphogenic events, Small Shelly Fossils are commonly phosphatized, yet preservation modes vary considerably—ranging from fine-scale replication as seen in some acrotretide brachiopods[1], to thin coating of skeletal walls as seen in Archaeocyaths[2], to the internal molds often associated with hyoliths[3]. These differences relate not only to the original composition of the skeletal structures, but also to fossil size and burial environment. Given this complexity, it is difficult to determine with certainty why the vertical skeletal walls are not visible in Fig. 1h. Nevertheless, the presence of skeletal walls in both taxa is well supported by the available evidence: specimens preserving only skeletal walls, specimens preserving only soft tissues, and critically, specimens preserving phosphatized soft tissues *in-situ* within skeletal chambers—all demonstrating that these structures were present when these organisms were alive. (Line 606–615)

Line 105: sac—sacs.
Response: Corrected and revised as suggested. (Line 108)

Line 121: muscles. (Fig. 1i----muscles (Fig. 1i
Response: Corrected and revised as suggested. (Line 124)

Line 122: The sacs---The sac membranes
Response: Corrected and revised as suggested. (Line 125)

Line 138: Front side --- Oblique front side
Response: Corrected and changed to "Oblique front side". (Line 142–143)

Line 143: three?

Response: We have removed the word “three” entirely to better explain the relevant features. The revised sentence is: Enlarged view of adjacent cystids. (Line 148)

Line 147: Hexagonal cystids with styles---Oblique view, showing hexagonal cystids with styles
Response: Revised as suggested. (Line 151–152)

Line 175: soft-part --- soft-tissue? May be consistent within the MS.
Response: Corrected and revised throughout the entire manuscript. (Line 181, 487)

Line 185: ‘the first of two independent biomineralization events in the Bryozoa’. Can this be briefly introduced?
Response: We have added additional text to the discussion to address this request, clearly indicating that, given both our new taxon and *Protomelission* represent putative stenolaemates, it is likely that they were both biomineralized. Unfortunately, because all of our specimens are secondarily phosphatized or silicified, it is impossible to be certain of their original composition. Two independent biomineralization events are currently recognized for the Bryozoa: the first in the Cambrian/Ordovician (Stenolaemata) and the second in the Jurassic (Cheilostomata). With evidence for biomineralized bryozoans now present in the Cambrian[4] (and in this study), this suggests the first biomineralization event took place in the early Cambrian, a result consistent with previous molecular work. (Lines 189–195)

Lines 351-352: ‘The phosphatized specimens were collected manually using acid maceration in 5–10% acetic acid’, this sentence does not well introduce clearly how to get the specimens. Actually, you may re-organize the paragraph ‘Material and occurrences’ for a clear knowledge!
Response: We have revised the sentences to follow a clearer logical flow covering the following areas: site and stratigraphy; acid maceration process; picking from residues; measurement methods. (Line 374–381)

Line 355: Cambrian Series 2, Stage 3---Cambrian Stage 3
Response: The indicated paragraph has been rewritten accordingly, and "Cambrian Series 2, Stage 3" has been revised to "Cambrian Stage 3" as suggested. (Line 375)

Lines 355-361: samples—specimens.
Response: Revised as suggested. (Line 385–388)

Line 502: arrows---arrow
Response: Corrected and revised as suggested. (Line 508)

Extended Data Figure 1C: what does it mean for the ‘stratigraphic horizon’ numbers? If they represent ‘beds or layers’, then the numbers should be put between the line bars. And the Xiaoyangba locality is also indicated in Figure 1b, but no specimens studied in this MS are from this locality.

Response: We confirm that the numbers represent stratigraphic horizons (beds/layers). Extended Data Figure 1C has been revised accordingly, with the numbers now placed between the lines as suggested. (Line 478, Extended Data Figure 1C)

The Xiaoyangba locality is included in Extended Data Figure 1b, as it is the locality from where *Protomelission gatehousei*, reported by Zhang et al.[5], was recovered. Although no specimens from this locality are described in the present study, we believe its inclusion provides useful context for readers familiar with the earlier discovery. We have now added reference to Zhang et al.[5] in the figure caption to clarify this relationship. (Line 482–484)

Extended Data Fig. 6: the outline differences of b and c both with a same scale should be briefly introduced, especially c with a same colour.

Response: We have revised the caption of Extended Data Fig. 6 (now Extended Data Fig. 3) to provide a clearer explanation. The updated caption now explicitly describes the differences between panels b and c, including the significance of the color coding, and clarifies the relationship between these panels. (Line 496–504)

'*P. gatehousei*, oligoserial colony form, tapering distally'. Is this form quite usual for bryozoans?

Response: The oligoserial colony form with a distally tapering morphology is indeed a common growth pattern for early stenolaemate bryozoans, particularly within certain cryptostome lineages. This colony architecture reflects modular addition during astogenetic development, where early-formed zooids establish the colony base with subsequent budding leading to distal tapering. Hageman et al.[6] defined an arrangement of zooecial series: "Oligoserial (3–12). Colonies that grow by budding several zooids along a growing margin are oligoserial. An upper limit of approximately 12 zooecial rows across the branch is placed on oligoserial colonies. In examples of bryozoan feeding behavior given in Winston[7,8,9] and McKinney[10], no zooecium is typically more than six zooecial positions away from an excurrent chimney or colony edge. This is the approximate width at which each zooid may still expel water from the edge of a flat colony without having to develop an integrated, communal feeding strategy. Oligoserial forms can be encrusting to erect, and are typically narrow strips, branches or cylinder." Similar colonial morphology has been documented in the Ordovician cryptostome bryozoan *Prophyllodictya gracilis*, in which branch thickness and width decrease from the colony base toward the distal end[11]. This feature supports our interpretation that the distally tapering colony form is consistent with the colonial growth pattern of bryozoans. Relevant notes regarding this issue have been added to the Systematic Palaeontology in Extended Data 1 (specifically the 'Remarks' section). (Line 594–596)

Orifice and aperture both being used. What is the difference? While in Extended Data Fig. 2 being orifice, it being aperture in Fig. 1J,K and in Diagnosis of new taxon.

Response: This is indeed an important terminological distinction. Past papers frequently used both terms in a random fashion. Such openings in the zooidal skeleton are conventionally referred to as apertures in stenolaemates, and orifices in

gymnolaemates[12]. The term orifice is used for the opening bordered by soft tissues and is, particularly in phylactolaemates and ctenostomes, synonymously used with the term aperture. In cyclostomes and cheilostomes, the term aperture is more commonly used for the skeletal aperture[13].

In our manuscript, we have adopted the following convention based on established bryozoan literature: **orifice refers to the opening bordered by soft tissues** (e.g., the membranous sac opening), while **aperture refers to the skeletal opening**. The skeletal aperture does not necessarily coincide with the orifice. We have carefully reviewed the entire manuscript and figure captions to ensure consistent usage and made clear in our systematic description the definitions outlined above. (Line 616–618)

Why vertical skeletal walls of adjacent zooids form a double wall (Extended Data Fig. 6f), while looking like a solid wall in Extended Data Fig. 3b?

Response: The apparent difference in wall structure between Extended Data Fig. 6f (now Extended Figure 3f) and Extended Data Fig. 3b (now Extended Figure 6b) reflects taphonomic variability in preservation modes. In most specimens, including those shown in Extended Data Fig. 3f, the skeletal walls are phosphatized, faithfully replicating the diagnostic double-wall structure formed by adjacent zooids, which is a common feature in post-Cambrian bryozoans. In contrast, the specimen in Extended Data Fig. 6b, while exceptionally preserving *in-situ* membranous sacs within the skeletal chambers, has undergone silica replacement of the skeletal walls, as confirmed by STEM-EDXS mapping (Extended Data Fig. 7). Although silicification can replicate morphology with reasonable fidelity, phosphatization is well known to preserve fine microstructural details more accurately. Thus, the observed difference in wall appearance is attributable to differential diagenetic mineralization pathways rather than any biological variation. (Line 506–508, 606–615)

Systematic Palaeontology: Why do not put the new taxon under the Class Stenolaemata if considering it as a crown group stenolaemate?

Response: This is a good suggestion given our results. Corrected and revised as suggested. (Line 556)

"Diagnosis—As for genus diagnosis by monotypy." This line should be under the new species name and above Description.

Response: We have now corrected the formatting in the Systematic Palaeontology section. The line "Diagnosis—As for genus diagnosis by monotypy" has been placed under the new species *Dayingomelission hexaclitia* gen. et sp. nov. and above the Description. (Line 573)

Within the Description paragraph, figure information may be cited for checking the features.

Response: We have revised the Description paragraph to include figure citations for key morphological features, allowing readers to directly verify each described character using the illustrated specimens. (Line 574–591)

For the new species, a holotype should be noticed.

Response: We have now ensured that the holotype and paratypes are clearly and separately indicated under the new species name, following standard palaeontological convention. (Line 568–570) and have also indicated in the relevant figure caption which specimen is the holotype (Fig. 2a–b).

Both taxa are with skeletal walls. How about the wall original composition?

Response: We thank Referee #1 for raising this excellent and important question regarding the original mineralogy of the skeletal walls. Determining the original composition of early Cambrian phosphatized Small Shelly Fossils is indeed a long-standing challenge and a topic of active research in the field. As demonstrated in recent studies (e.g., Li et al.[14,15,16,17]), well-preserved microstructures can sometimes provide clues for inferring original mineralogy in certain groups such as molluscs. In our material, although the specimens are exquisitely preserved, the skeletal walls have been entirely replaced by phosphate or silica during diagenesis, and the microstructural details preserved are insufficient to confidently determine their original composition. That the original skeletal mineralogy his unknown is now stated in the manuscript. (Line 190–193)

However, this uncertainty does not affect our core conclusions. The presence of skeletal walls in life is unequivocally demonstrated by multiple lines of evidence (e.g., styles and ring septa). We hope that future discoveries of even better-preserved material may shed light on the original mineralogy of early Cambrian bryozoans, but we emphasize that this does not diminish the significance of our findings regarding the Cambrian origin and early diversification of bryozoans.

Referee #2 and #4 (Remarks to the Author):

Song et al. present compelling evidence supporting the Cambrian origin of the Bryozoa. They found phosphatized fossils of two bryozoan taxa from an early Cambrian formation of China. Their results are original and of great significance, because two studies (Yang et al., 2023, Nature 615: 468–471; Xiang et al., 2024, Geology 52: 130–134) published after their original finding (Zhang et al., 2021, Nature 599: 251–255) casted doubts about the bryozoan nature of this fossil.

They based their conclusions on a variety of techniques, such as Scanning Electron Microscopy, focused ion beam-Transmission Electron Microscopy, X-ray computed tomography and STEM-EDXS mapping. Phylogenetic inferences were based on Bayesian Analysis of a matrix including Protomelission and Dayingomelission, 18 bryozoan genera, two outgroups and 50 characters.

Their conclusions are robust and reliable, since they provide images of soft tissues (e.g., membranous sacs) preserved in-situ within modular structures and typical bryozoan structures such as styles. The Abstract and the Introduction are complete and appropriate, introducing the reader to fundamental studies on the subject.

Response: We thank Referee #2 and Referee #4 for their positive and encouraging assessment of our work.

The Reference list is exhaustive and up-to-date, although I found some minor problems that should be corrected before acceptance: (1) Delete the comma after the journal name on line 264. (2) Replace the colon with a period on line 266. (3) Marcusodictyon instead of Marcusodictyan on line 273, with italics. (4) Incomplete title of articles on lines 269 ("Taxonomic notes on genera of fossil and recent Bryozoa") and 276-277 ("The oldest bryozoans: new evidence from the Late Tremadocian (Early Ordovician) of East Yangtze Gorges in China)". (5) Incomplete journal name on line 245. (6) Begin with a capital letter after the author's name on line 257. (7) On line 292, the author's initials are M.A.S and in "The Cambrian Explosion:" each word begins with a capital letter. (8) The family name of the second author on line 294 is Wyse Jackson. (9) DOIs should be added whenever possible. (10) Abbreviate journal names on lines 266–267, 268, 269–270, 293, 313, 315–316, 318, 332–333, 336 and 339.

Response: All the minor issues noted have now been addressed and corrected in the revised manuscript. (Line 263, 270, 275, 282, 284–285, 286, 288–289, 292, 295, 296, 311–312, 334, 335–337, 339, 345, 348, 353–354, 357–358, 361)

Following the *Nature*'s reference formatting guidelines, we have added the DOI for Song et al.[1] (Line 358), which is a recently published online article without volume and page numbers. Whilst providing DOIs for all papers is a good suggestion, for other journal articles and books, the Nature reference format does not indicate DOIs should be included, and we have consistently followed this format throughout the manuscript.

Referee #3 (Remarks to the Author):

The manuscript describes a new Cambrian bryozoan taxon, and provides further information on a previously known species. The fossils are exceptionally well preserved and features of the skeleton make it clear that the material belongs to the Phylum Bryozoa and the conclusions are justified. This also resolves the contention by some authors that the species described by Zhang 2021 was erroneously assigned to bryozoans. The presence of two distinct species, with differing morphotypes. in the Cambrian also suggests a common ancestor in the early Cambrian or even Ediacaran, and this interpretation is justified. However the text is inconsistent with respect to wall mineralogy (see below for details). An interpretation is made that these biomineralized walls represent an independent mineralisation event and the authors suggest a common ancestor earlier. This suggestion of an earlier ancestor, which is justified based on two taxa with different bryozoan morphotypes, does not hinge on biomineralization. The significance of the manuscript does not depend on being able to confirm biomineralization during life, but the authors interpretation does need to be clarified.

The methodology is appropriate and the figure presentation is of good quality (noted in the annotated pdf some figure captions need species presented defined), and the references are appropriate. The abstract is clear and appropriate to the

content of the manuscript.

This is a significant contribution and is worthy of publication.

Response: We sincerely thank Referee #3 for their thorough and constructive evaluation of our manuscript, and for their positive assessment of its significance and recommendation for publication.

Regarding the specific point about wall mineralogy and biomineralization, we fully agree that clarification is needed. We note that this question and the following question address the same issue. To avoid repetition, we will provide a unified response in our reply to the next comment.

ADDITIONAL COMMENTS (see also the annotated pdf)

Wall mineralogy

Be clear about whether the fossils were phosphatised after death as a diagenetic process, or do you mean to say they had a phosphatic skeleton during life stages. I think you mean the former (and this was stated in the Zhang et al original description of P. gatehousei) but in places this is not clear in your manuscript. I have tried to highlight these in the annotated pdf.

The terminology in the results for both taxa refer to phosphatisation or phosphatised fossil/walls/structures – ie the phosphatisation occurred after death of the bryozoan. However in the discussion at line 183-186 it is stated that this is the first evidence of biomineralized stenolaemates, which indicates you are interpreting that they were biomineralized in life. This conflicts with the terminology given in the results.

Phosphatised wall = the wall is replaced not original mineralogy

Phosphatic wall = the wall was made of phosphate by the organism, or replaced.

The elemental maps in Extended Data Fig 2 only show that phosphate and silica are present, they do not confirm when this happened (during life or after death). So the intention of the authors needs to be made clear in the text.

What is the evidence that these fossils were made of phosphate during life? The diagenetic replacement process could result in perfect replication of an original organic wall, so if you think these were mineralised during life a case needs to be made for that. This is not to suggest that Cambrian bryozoans were not phosphatic walled, but evidence needs to be supplied or an argument made. To my knowledge the lingulids are still considered the first brachiopods, and they were phosphatic (and still are), so that bryozoans may have first been phosphatic is not out of the question. But an argument needs to be made for this.

Response: We thank Referee #3 for this incisive and constructive critique regarding wall mineralogy. We fully agree with the reviewer's assessment and have addressed the concerns as follows:

Terminology clarification: We have carefully revised the entire manuscript to ensure consistent use of terminology. "Phosphatized" is now used exclusively to indicate diagenetic replacement post-mortem, consistent with the original description of *P. gatehousei* by Zhang et al.[5]. All instances where the text previously implied we were referring to the primary composition of our specimens have been corrected to

make clear we are referring to secondary replacement by phosphatic material. It is common in studies of early Cambrian fossils to use the term 'phosphatized' to refer to material that has been secondarily replaced, but we concede in hindsight that this is not a broadly understood convention, and have clarified the text appropriately.

Original mineralogy: We acknowledge that the current evidence—including the elemental maps in Extended Data Fig. 5—only confirm the presence of phosphate and silica in the fossil specimens, but cannot determine whether the original skeleton was phosphatic, calcitic, or even organic during life. We have therefore refrained from speculating on original mineralogy and have revised the discussion accordingly (Lines 188–192) to avoid any implication that these represent primary phosphatic skeletons.

All of the annotations in the provided PDF have been carefully reviewed and the text revised to ensure terminological consistency and clarity. (Line 28, 30, 68, 75, 89, 119, 189–193, 505–508, 518–519, 527, 539–540, 545, 606–615).

Siliceous replacement is likely spherulitic and some of your specimens have been repalced with silica. What is the mineral composition of the specimen in 2i? Are you interperting it as replacement?

Response: We thank Referee #3 for this specific observation regarding silica replacement. The specimen shown in Fig. 2i is predominantly phosphatized, not silicified. As noted in the manuscript, silicified preservation occurs only in a subset of our material, such as the specimen shown in Fig. 3c–g. For the vast majority of specimens, including Fig. 2i, the dominant preservation is phosphatization.

Systematic Palaeontology – no holotype is defined in the description and the measurement tables do not have units, or identification of what structure is being measured, and what each line refers to. This must be corrected. The systematic description of *D. hexaclitia* should be in the extended data section.

Response: We have now explicitly designated the holotype and paratypes for *Dayingomelission hexaclitia* gen. et sp. nov. with full repository information, revised the measurement tables to include clear units (μm) and structure identification, and moved the complete systematic description to the Extended Data section as suggested. (Line 509–516, 568–570, Extended Data Fig. 4, Supplementary Data 1 Measurement of *Protomelission gatehousei* and *Dayingomelission hexaclitia* gen. et sp. nov.).

References cited

1. Song B. et al. Small skeletal fossils associated with archaeocyath reefs from the Xiannüdong Formation (Cambrian Series 2), southern Shaanxi, China. *Palaeobio. and Palaeoenv.* (2026). https://doi.org/10.1007/s12549-025-00677-y
2. Wang, J., Song, B., Liang, Y., Liang, K. & Zhang, Z. The Internal Anatomy and Water Current System of Cambrian Archaeocyaths of South China. *Life* **14**, 167 (2024).
3. Hu, Y. et al. Preservation potential of Cambrian small shelly fossils in different microfacies, North China. *Geosci. Front.* **16**, 102108 (2025).

4. Pruss, S. B., Leeser, L., Smith, E. F., Zhuravlev, A. Y. & Taylor, P. D. The oldest mineralized bryozoan? A possible palaeostomate in the lower Cambrian of Nevada, USA. *Sci. Adv.* **8**, p.eabm8465 (2022).
5. Zhang, Z. et al. Fossil evidence unveils an early Cambrian origin for Bryozoa. *Nature* **599**, 251–255 (2021).
6. Hageman, S. J., Bock, P. E., Bone, Y. & Mcgowran, B. Bryozoan growth habits: Classification and analysis. *J. Paleontol.* **72**, 418–436 (1998).
7. Winston, J. E. Feeding in marine bryozoans. In: R. M. Woollacott, and R. L. Zimmer (eds.), *Biology of Bryozoans*. Academic Press, London, 233–271 (1977).
8. Winston, J. E. Current-related morphology and behaviour in some Pacific coast bryozoans. In: G. P. Larwood, and M. B. Abbott (eds.), *Advances in Bryozoology, Systematics Association Special Volume, 13*. Academic Press, London, 247–268 (1979).
9. Winston, J. E. Feeding behavior of modern bryozoans In: T. W. Broadhead (ed.), *Lophophorates: Notes for a Short Course*. University of Tennessee Department of Geological Sciences Studies in Geology, 1–21 (1981).
10. McKinney, F. K. Evolution of Erect Marine Bryozoan Faunas: Repeated Success of Unilaminate Species. *The American Naturalist* **128**, 795–809 (1986).
11. Key, M. M., Håkansson, E. & Edwards, B. R. Functional controls on monticule height and spacing in Permian stenolaemate bryozoans. *J. Paleontol*. **98**, 959–972 (2024).
12. Taylor, P. D. *Bryozoan Paleobiology*. (John Wiley & Sons, 2020).
13. Schwaha, T. *Phylum Bryozoa, Handbook of Zoology*. (De Gruyter, 2020).
14. Li, L., Zhang, X., Yun, H. & Li, G. Complex hierarchical microstructures of Cambrian mollusk *Pelagiella*: insight into early biomineralization and evolution. *Sci. Rep.* **7**, 1935 (2017).
15. Li, L. et al. Shell microstructures of the helcionelloid mollusc *Anabarella australis* from the lower Cambrian (Series 2) Xinji Formation of North China. *J. Syst. Palaeontol.* **17**, 1699–1709 (2019).
16. Li, L., Skovsted, C. B. & Topper, T. P. Deep origin of the crossed-lamellar microstructure in early Cambrian molluscs. *Palaeontol.* **65**, e12620 (2022).
17. Li, L. et al. Fibrous or Prismatic? A Comparison of the Lamello-Fibrillar Nacre in Early Cambrian and Modern Lophotrochozoans. *Biology* **12**, 113 (2023).

Response to Reviewers' Comments

Referee #1 (Remarks to the Author):

In this revised MS, the authors answered all our quesions and accepted most of our reviewers' suggestions. Great work. I am happy with their work!
I nearly have no more suggestions, but for Line 198: 'early Cambrian' may be 'early Terreneuvian' or 'early Cambrian (Terreneuvian)' since the described fossils are also described as the early Cambrian in the MS.
Response: We thank Referee #1 for your strong recommendation for publication in *Nature*. We have revised the sentence accordingly: These results also imply that the common ancestor of the organic-walled Gymnolaemata and the mineralized Stenolaemata likely originated in the early Cambrian (Terreneuvian) or even perhaps in the Ediacaran period[21,23,38]. Line 141.

Referee #2 (Remarks to the Author):

The authors have addressed all the requested changes. The manuscript may now be published as it is.
Response: We thank Referee #2 for your recommendation for publication in *Nature*.

Referee #3 (Remarks to the Author):

Dear Zhifei Zhang,
Thank-you for your response to my comments regarding wall mineralogy. It is now clear from the text that the fossils were phosphastised after death and that the composition of the skeleton in live remains unknown. Thank-you for moving the systematic palaeontology to the extended dxata section where it will remain a part of the archived article. These changes address my key concerns on the original manuscript and I support publication in its current form.
Regards
Catherine Reid
Response: We thank Referee #3 for your strong recommendation for publication in *Nature*.

Referee #4 (Remarks to the Author):

I co-reviewed this manuscript with one of the reviewers who provided the listed reports.
Response: We thank Referee #4 for your recommendation for publication in *Nature*.
